# Membrane Mediated Antimicrobial and Antitumor Activity of Cathelicidin 6: Structural Insights from Molecular Dynamics Simulation on Multi-Microsecond Scale

**DOI:** 10.1371/journal.pone.0158702

**Published:** 2016-07-08

**Authors:** Bikash Ranjan Sahoo, Toshimichi Fujiwara

**Affiliations:** Laboratory of Molecular Biophysics, Institute for Protein Research, Osaka University, Suita, Osaka, Japan; Bose Institute, INDIA

## Abstract

The cathelicidin derived bovine antimicrobial peptide BMAP27 exhibits an effective microbicidal activity and moderate cytotoxicity towards erythrocytes. Irrespective of its therapeutic and multidimensional potentiality, the structural studies are still elusive. Moreover, the mechanism of BMAP27 mediated pore formation in heterogeneous lipid membrane systems is poorly explored. Here, we studied the effect of BMAP27 in model cell-membrane systems such as zwitterionic, anionic, thymocytes-like (TLM) and leukemia-like membranes (LLM) by performing molecular dynamics (MD) simulation longer than 100 μs. All-atom MD studies revealed a stable helical conformation in the presence of anionic lipids, however, significant loss of helicity was identified in TLM and zwitterionic systems. A peptide tilt (~45˚) and central kink (at residue F10) was found in anionic and LLM models, respectively, with an average membrane penetration of < 0.5 nm. Coarse-grained (CG) MD analysis on a multi-μs scale shed light on the membrane-dependent peptide and lipid organization. Stable micelle and end-to-end like oligomers were formed in zwitterionic and TLM models, respectively. In contrast, unstable oligomer formation and monomeric BMAP27 penetration were observed in anionic and LLM systems with selective anionic lipid aggregation (in LLM). Peptide penetration up to ~1.5 nm was observed in CG-MD systems with the BMAP27 C-terminal oriented towards the bilayer core. Structural inspection suggested membrane penetration by micelle/end-to-end like peptide oligomers (carpet-model like) in the zwitterionic/TLM systems, and transmembrane-mode (toroidal-pore like) in the anionic/LLM systems, respectively. Structural insights and energetic interpretation in BMAP27 mutant highlighted the role of F10 and hydrophobic residues in mediating a membrane-specific peptide interaction. Free energy profiling showed a favorable (-4.58 kcal mol^-1^ for LLM) and unfavorable (+0.17 kcal mol^-1^ for TLM) peptide insertion in anionic and neutral systems, respectively. This determination can be exploited to regulate cell-specific BMAP27 cytotoxicity for the development of potential drugs and antibiotics.

## Introduction

Small cationic peptides that possess cell penetrating ability are a component of natural immune systems and comprise a large family of immune-defense molecules found in most living organisms [1, 2]. The antimicrobial and anticancer activities are mediated by a series of steps that include peptide attraction to the membrane, binding, distribution/aggregation, reorientation and insertion followed by cell lysis [3]. These peptides have cationic and hydrophobic amino acid residues and assume variable secondary structures such as α-helix or β-strands with disulfide bonds or extended conformation [4, 5]. The membrane permeability and disruption greatly depend on their secondary structures, amino acid compositions and the membrane environment [6–8].

The neutrophil-derived cathelicidin 6 (BMAP27) found in bovine is a 17.8 kDa protein and is characterized by an N-terminal cathelin domain and C-terminal active peptide [9, 10]. The active peptide is composed of 27 residues (GRFKRFRKKFKKLFKKLSPVIPLLHLG) and exhibits microbicidal activity towards Gram-negative, Gram-positive bacteria and fungi cells. At higher concentrations significant cytotoxic effect towards human erythrocytes and neutrophils has also been reported [9, 11]. Structurally, BMAP27 is characterized by an amphipathic α-helix in the N-terminus and a hydrophobic extended loop at the C-terminus. Its helical conformation was studied by circular dichroism (CD) and solution nuclear magnetic resonance (NMR) techniques [9, 12]. Truncation of the hydrophobic C-terminal end has been shown to cause a significant decrease in the peptide cytotoxicity to human erythrocytes and neutrophils, while conserving their microbicidal activity [9, 11, 12]. BMAP27 is moderately cytotoxic to human neutrophils and erythrocytes [12]. Their depolarization potentiality at the inner mitochondrial membrane followed by cell death suggests its membrane permeability [11]. In spite of the relevance of their dual cytotoxic activity towards microbial and tumor cells [10, 12], the structural characterization of these peptides is still insufficient. Due to an urgent need of developing novel drugs and antibiotics against drug-resistant pathogens and deadly tumor cells, the moderate cytotoxicity of BMAP27 can be optimized to develop potential drugs. The therapeutic applications and development of such peptides have been a large topic of many computational simulations [13, 14, 15] and NMR studies [8, 16–17]. However, the structures in bovine cathelicidins have not been studied so far.

Several models such as carpet model, barrel-stave model, toroidal pore model, etc. have been proposed to understand the mechanism of membrane pore formation. However, a cogent experimental insight for the mechanism of the cell disruption remains elusive. These proposed models also vary across the AMPs and depend on peptide secondary structure, sequence composition, sequence length, membrane lipid compositions etc. [3, 18–20]. The helix-loop arrangement in BMAP27 with a substantial charged helix and hydrophobic loop indicate its potential membrane-perturbing activity. Previously, several computational studies have been carried out for other α-helical peptides using all-atom and coarse-grained (CG) molecular dynamics (MD) simulation in homogeneous or heterogeneous lipid bilayer membranes to explore the mechanism of AMP action [21–23]. Most of the studies were carried out on a nanosecond time scale and few studies included calculations on a microsecond scale. However, to our knowledge, no structural studies have been performed for BMAP27. Moreover, the interaction mechanism of this AMP with lipid bilayers mimicking the membrane of cancer cells has not been explored yet.

Here, we studied the membrane interaction of BMAP27 and its analogue in different model membranes such as anionic, zwitterionic, thymocytes-like (TLM), leukemia-like (LLM) and hypothetical proliferative-like (hyPLM) membrane. The limitations in previous MD simulation studies such as small time scale simulation, biased MD (steered MD and high temperature mediated peptide transition), artificial pore formation, etc. were warded off by performing unbiased all-atom and CG-MD simulations on time scales longer than 100 μs in homogeneous and complex heterogeneous lipid environments. Comparative study of BMAP27 in TLM and LLM systems highlighted differential modes of peptide binding, oligomerization, bilayer reorganization and membrane permeability efficacy. Membrane dependent BMAP27 folding, oligomerization and kinetics were parameterized concerning their mechanism of actions in membrane surface. Potential of mean force profiling was performed to monitor the energetic favorable and unfavorable membrane systems for BMAP27 insertion using umbrella sampling technique. These results can be utilized to develop BMAP27 analogues with enhanced cytotoxicity against bacteria and tumor cells.

## Materials and Methods

### Simulation systems

The initial 3D coordinates of BMAP27 determined in a solution were retrieved from the PDB database (PDB ID: 2KET) and the lowest energy structure was considered for structural interpretation. MD simulation of the initial structure in the pure solvent medium was carried out to analyze their secondary structural properties in the absence of lipid membrane environments in AMBER99SB-ILDN [24] and CHARMM36 [25] force fields for a time period of 0.5 μs. Anionic and zwitterionic lipid bilayers were created using MemBuilder [26] program and were equilibrated prior to peptide addition. The equilibrated lipid bilayer systems were considered for peptide-lipid structural analysis using Slipid/AMBER and AMBER99SB-ILDN force fields for lipid and peptide, respectively. A peptide to lipid (P/L) ratio of 1:128 was considered to study the peptide effects on membranes at moderate concentration. The exact P/L required for pore formation and membrane disruption is still controversial. Therefore, much higher (1:25) and much lower (1:200) concentration was avoided for the all-atom MD simulation. To investigate the peptide orientation in an anionic environment, additional MD calculation was performed on a dioleoylphosphatidylglycerol (^sp^DOPG) lipid-bilayer system. In this simulation the BMAP27 molecule was partially inserted into the outer leaflet (~10 Å from bilayer surface) with the peptide helix oriented parallel to the membrane surface and the C-terminal facing the polar solvents using *g_membed* program [27].

The asymmetric TLM, hyPLM and LLM systems comprised of 128 lipid molecules and one BMAP27 peptide were modeled using the CHARMM-GUI membrane builder [28]. The different molar composition of lipid molecules in the plasma membrane of murine thymocytes and leukemia cells were determined by reference to the previous reports [29] and modeled as described elsewhere [30]. The molar concentration of cholesterol to phospholipids (~ 42.5% in TLM and 24.2% in LLM) were carefully accounted for the lipid system generation [30]. The heterogeneous systems such as TLM, LLM and hyPLM were composed of phosphatidylcholine (PC), phosphatidylethanolamine (PE), phosphatidylserine (PS), phosphatidyl-myo-inositol (PI), sphingomyelin (SM), lyso-PC, lyso-PE and cholesterol. The 5.3% (7 PS molecules) anionic phospholipids were distributed along the inner leaflet of TLM. On the other hand, 7.4% (10 lipids) of PS molecules were arranged along the outer leaflet of LLM. The hyPLM was created to mimic a proliferative cell membrane where a part of the anionic PS lipids were exposed to the outer surface by amino-phospholipid translocase activity [31]. The lipid compositions of hyPLM system was same as TLM [30] with a little increase in PS concentration (6.2%) for symmetric distribution across the outer and inner membrane surfaces. The symmetric PS distribution provided a surface charge to the hyPLM model membrane with 3.1% anionic lipids on the exofacial surface. The CHARMM36 and AMBER99SB-ILDN force fields were best considered for our MD calculations due to the recent improvements for peptide folding and unfolding analysis [32]. The different MD systems are detailed in Table 1.

**Table 1:** Summary of molecular dynamics simulation systems.

MD systems	Force field	Lipid no.	Peptide no.	Solvent	Time (μs)
All-atom MD					
Solvent	AMBER99SB-ILDN	0	1	5675	0.5
Solvent	CHARMM36	0	1	5652	0.5
POPC	AMBER99SB-ILDN	128	1	9395	0.5
DOPG	AMBER99SB-ILDN	128	1	7689	0.5
^sp^DOPG	AMBER99SB-ILDN	126	1	7896	0.1
TLM	CHARMM36	128	1	3306	0.5
LLM	CHARMM36	128	1	3603	0.5
DOPG-BMAP27_mut_	CHARMM36	128	1	7906	0.25
LLM-BMAP27_mut_	CHARMM36	128	1	3696	0.25
**Coarse-grain MD**					
DPPC	MARTINI	226	4	3830	10
DOPG	MARTINI	430	4	8577	10
DOPS	MARTINI	226	4	4968	10
Solvent	MARTINI	0	4	12518	2
LLM	MARTINI	226	4	5096	20
hyPLM	MARTINI	226	4	5154	20
TLM	MARTINI	226	4	5142	20
Carpet-like oligomer	MARTINI	1946	20	72811	13
Micelle-like oligomer	MARTINI	252	10	4902	10
DPPC/trBMAP27^1-18^	MARTINI	226	4	4072	10
DOPS/trBMAP27^1-18^	MARTINI	226	4	3696	10

TLM, thymocytes-like membrane; LLM, leukemia-like membrane; hyPLM: hypothetical proliferative-like membrane; trBMAP27^1-18^, truncated BMAP27 (1–18).

### MD parameters

All-atom MD simulations were carried out using GROMACS MD software package [33], version 4.5.5 and 5.0.2 running parallel with SGI UV 3000 at Institute for Protein Research, Osaka University, Japan. Standard force field parameters were considered for the peptide-lipid MD systems. The cationic BMAP27 molecule with +10 charge was neutralized by counter Cl^-^ ions, and a salt (NaCl) concentration of 100 mM in the TIP3P solvent medium was considered for MD simulation (Table 1). The peptide was initially placed at ~15 Å away from the outer leaflet of the lipid bilayer. The simulation systems were maintained at 310K temperature for peptide, ions and water molecules. The lipid temperature was chosen above the phase transition temperature for homogeneous systems, and 323K was considered for heterogeneous systems using the Nose-Hoover thermostat [34, 35] and a time constant of 0.1 ps. One bar semi-isotropic pressure coupling with a time constant of 5 ps using the Parrinello-Rhaman barostat [36] conditions were applied. Electrostatic interactions were calculated using the particle mesh Ewald algorithm with a coulomb cutoff of 10 Å, Fourier spacing of 1.2 Å and interpolation order of four. A cutoff distance of 12 Å was used for non-bonded interactions. LINCS algorithm was used to constrain all bonds involving hydrogen atoms. MD simulations were carried out in constant particle number, pressure, and temperature ensemble with peptide, lipid, water and ions coupled separately. Simulations were run at a time step of 2 fs and the neighbor lists were updated in every five steps. All-atom MD simulation was also performed in the BMAP27_mut_ molecule with central kink residue mutation (F10 –A10).

The CG-MD simulations were also performed using GROMACS software package in parallel using MARTINI CG force field [37]. The CG models of the peptide and lipid molecules were generated using the MARTINI models and the structural properties were restrained. The NMR structure of BMAP27 was considered and the helical content was fixed. The homologous human cathelicidin was shown to form a helix structure at a higher concentration (P/L = 1:50 or 1:20) in both bacterial and eukaryotic systems as evidenced from the CD experiment [38]. Therefore, referring to the experimental evidences in BMAP27 homologous and other helical AMPs [38, 39], we selected the NMR conformation for our analysis. A 4:1 mapping of (non-hydrogen) atoms onto CG particles was considered for all CG approaches. Different lipid bilayer systems were created using the INSANE python program. The P/L compositions are listed in Table 1. Each bilayer was equilibrated for 1 μs before the addition of the peptide. The BMAP27 peptide molecules were placed far away from the lipid bilayer by ~ 20–30 Å with different orientations. Comparative analysis between TLM and hyPLM was carried out to investigate the peptide binding and oligomerization affinity at equal and unequal cholesterol and 1,2-dioleoyl-*sn*-glycero-3-phosphoserine (DOPS) distributions, respectively. Two artificial initial BMAP27 oligomers in micelle and end-to-end arrangements were designed with a variable P/L ratio to interpret their membrane penetration ability and oligomerization stability efficacy. An integration time step of 20 fs for the production MD run, and non-bonded interaction cutoff of 1.2 nm were used for all CG-MD simulations. The coulomb and Lennard-Jones potentials were smoothly shifted to zero in the range of 0–1.2 nm, and 0.9–1.2 nm, respectively. The peptide, lipids, water molecules and ions were independently coupled. The temperature was referenced from our all-atom MD simulation by applying velocity rescaling thermostat, and 1 bar semi-isotropic pressure was weakly coupled using Parrinello-Rahman barostat. Periodic boundary condition and standard non-bonded interaction were applied to all systems. The membrane thickness was calculated using GridMAT-MD program [40] for the equilibrated membrane (without BMAP27) and the simulated membrane (with BMAP27) for comparative analysis. The thickness between the outer and inner leaflets was measured and plotted using gnuplot (http://www.gnuplot.info/) in a 20 x 20 matrix. Prediction of hot spots of BMAP27 aggregation was performed using the AGGRESCAN [41] program from the primary amino acid sequence derived from UniProt (ID: P54228) database.

MD trajectories were examined and visualized with Visual Molecular Dynamics (VMD) program version 1.9.2 [42], and interpreted using GROMACS program. 3D visualization of peptide and lipid molecules was accomplished using VMD, PyMOL (http://www.pymol.org/) and Accelrys^®^ Discovery Studio 3.5 Visualizer. The 2D graphs were generated using Grace (http://plasma-gate.weizmann.ac.il/Grace/), and gnuplot programs.

### Free energy calculations

To explore the binding kinetics of BMAP27 into model lipid bilayers, we estimated the potential of mean force (PMF) in POPC, DOPG, TLM and LLM systems. The CG-MD model was selected for the PMF analysis and has been implemented for other α-helix protein analysis [43, 44]. The PMF was calculated using the umbrella sampling protocol in GROMACS. The BMAP27/ BMAP27_mut_ molecule was initially set at a distance of ~ 5 nm away from the center of the bilayer (z = 0). A force constant of 1 kJ mol^-1^ nm^-2^ was applied to the center of mass (COM) of BMAP27 and pulled along the z-axis in perpendicular to the membrane surface. Overall, 75 different peptide distances from the center of the bilayer were sampled, and equilibrated for 25 ns. A final production run of 75 ns was employed using biasing potential to restrain the center of mass of peptide followed by PMF profiling through weighted histogram analysis method [45].

## Results

### All-atom MD simulation

#### Structural analysis of BMAP27 in aqueous and homogenous lipid systems

**Fig 1:**
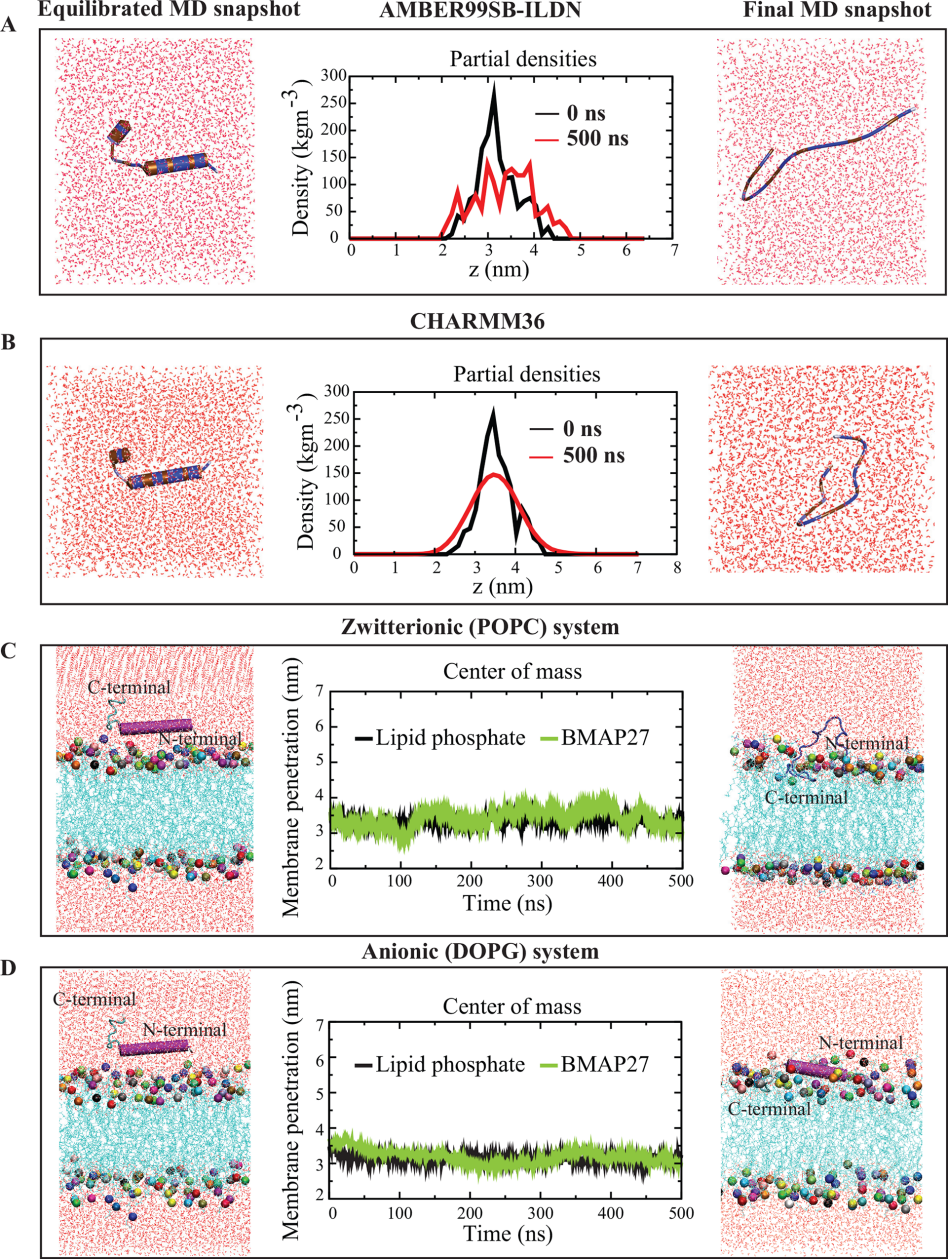
Conformational analysis of BMAP27 in aqueous and model lipid-bilayer environments. BMAP27 folding studied in (A) AMBER99SB-ILDN, and (B) CHARMM36 force field on a 0.5 μs time scale. The peptide and solvent molecules are represented as schematic and lines in Discovery studio visualizer 3.5, respectively. The peptide is colored by its hydrophobicity where hydrophobic and hydrophilic residues are shown as brown and blue, respectively. The partial density changes across the corresponding simulation boxes are shown in the central column. The z-coordinate represents the peptide position inside the simulation box. BMAP27 interaction with (C) zwitterionic (POPC), and (D) anionic (DOPG) membrane model systems. The peptide conformation is shown as a cartoon, lipid in blue line and water as red lines in VMD program. The relative BMAP27 membrane penetration is calculated by plotting the center of mass of peptide and lipid head phosphates present in the outer leaflets.

Secondary structure of BMAP27 in aqueous medium and force field dependent peptide folding was examined. BMAP27 in aqueous solution displayed an extended conformation and occupied a large conformational space in both force fields (Table 1) during the 0.5 μs MD simulation (Fig 1A and 1B). The partial densities of the peptide distribution were broadened and ensured an unfolded BMAP27 conformation (Fig 1A and 1B).

Simulations for BMAP27 interaction with zwitterionic and anionic membranes depicted a differential peptide folding and kinetics. Results showed a fast peptide attraction (~ 50–80 ns) followed by slow binding and reorientation (~100–500 ns) on the lipid-bilayer surface. The calculated α-helical conformation of BMAP27 revealed a significant helix loss (Fig 1C) in the 1-palmitoyl-2-oleoylphosphatidylcholine (POPC) system at the end of the 0.5 μs simulation. By contrast, the helicity was well restrained in 1,2-dioleoyl-*sn*-glycero-3-phospho-rac-1-glycerol (DOPG) system (Fig 1D). The MD results of BMAP27 correlated the experimental findings that showed a folded and unfolded conformation for the homologous human cathelicidin (LL-37) protein in zwitterionic and anionic membranes, respectively [38]. To our knowledge, a well-defined hydrophobic movement and amphipathicity in short peptides defines their helix stability through intermolecular and/or intramolecular interaction. Thus, the unfolded and membrane associated state of BMAP27 in zwitterionic systems indicated an unfavorable peptide amphipathicity. In addition, the helical propensity could also depend on the P/L that has been limited in our calculation.

The secondary structure profiling analysis in VMD revealed a significant structural change in zwitterionic systems as compared to anionic model membrane systems (S1A and S1B Fig). An irregular and inverted U-shaped BMAP27 conformation was observed in the POPC lipid system with the helix region orienting away from the membrane surface (Fig 1C). Small helical tilt and comparatively deep membrane penetration along the N-terminus was identified in the DOPG model systems. The tilt analysis in the ^sp^DOPG system further displayed an apparent peptide tilt angle of ~ 45° to the bilayer normal with its N- and C-termini located on the membrane surface and in the hydrophobic core, respectively (S2A Fig). The COM analysis for the comparison between the peptide and lipid heads in the two membrane models indicated the adsorption and penetration of the peptide by less than 0.5 nm during the 0.5 μs simulation (Fig 1C and 1D).

**Fig 2:**
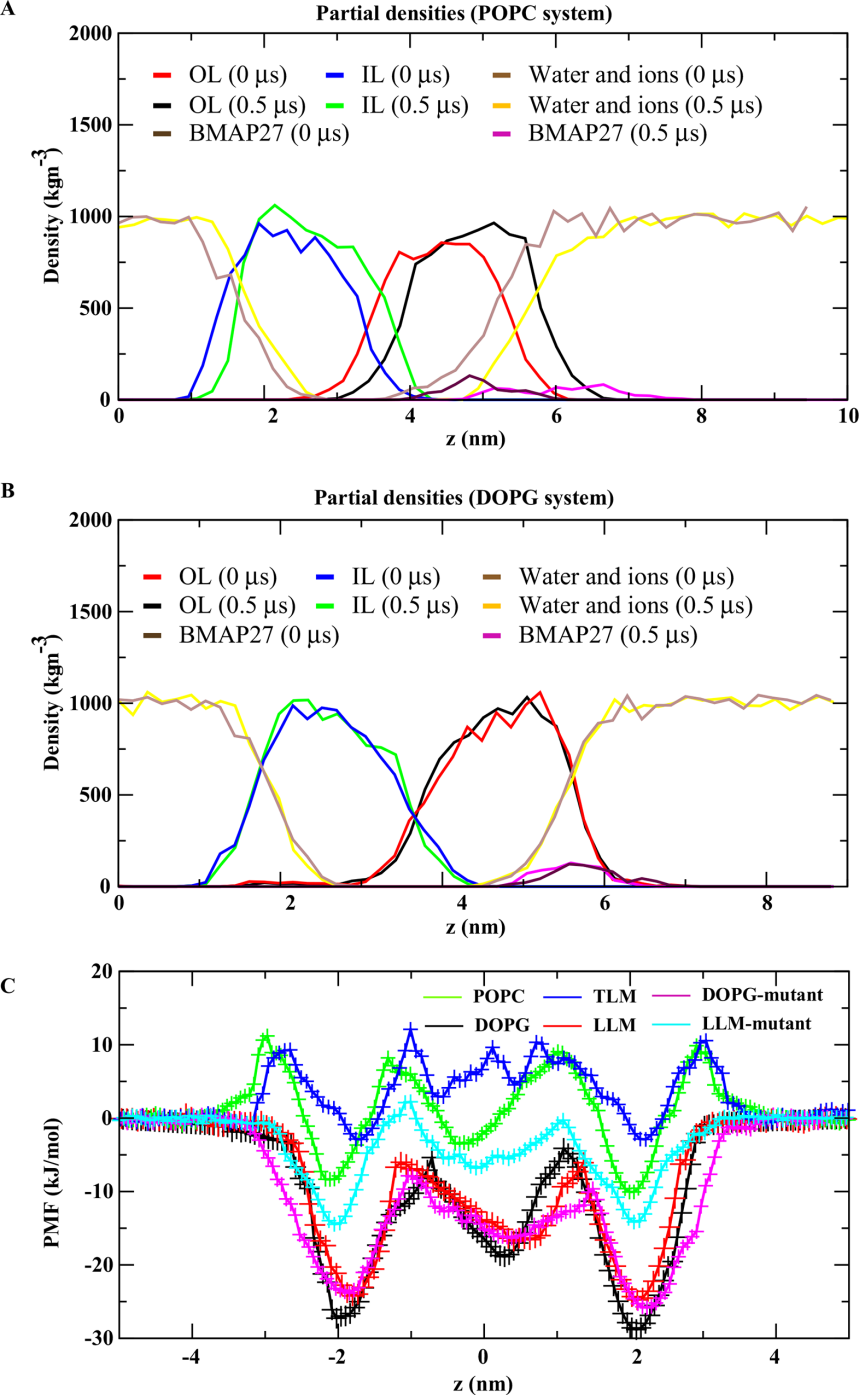
Partial density analysis of peptide, lipid, water and ions in different lipid-bilayer systems. (A) POPC, (B) DOPG membrane system. The outer and inner leaflets of the bilayer membrane are denoted as OL and IL, respectively. The contribution of various types of atoms are shown in the graph in different colors during the 0.5 μs MD simulation. (C) Potential of mean force acting on the BMAP27 to penetrate the lipid bilayer membrane. The horizontal axis shows the distance between the peptide and lipid bilayer membrane with the bilayer core situated at z = 0. The peptide molecule insertion path is perpendicular to the bilayer surface. The PMF calculation in BMAP27_mut_ system is carried out only for the anionic systems and are compared with the PMF values of wild type BMAP27.

In the POPC system, the peptide interacted with lipids majorly at two N-terminal cations (R2 and R5), and other helical cationic residues showed no hydrogen bond (H-bond) interaction (Table 2). On the contrary, a relatively large number of H-bonds were depicted in DOPG system. The H-bonds in DOPG system were majorly contributed by the N-terminal arginine (R2, R5 and R7) and lysine (K8 and K9) residues (Table 2). The mass density profile of the both systems depicted a little variation in the bilayer thickness. The change in thickness was < 5 Å, and a comparatively large water and lipid density change was obtained from the POPC system. The effect of BMAP27 binding on membrane dynamics of the two lipid systems was compared using their average lateral diffusion coefficient (D_L_) derived from the initial (0–10 ns) and final (490–500 ns) 10 ns time period. In the POPC system, the average D_L_ of 0.016 (± 0.003) x 10^−5^ cm^2^ s^-1^ and 0.008 (± 0.004) x 10^−5^ cm^2^ s^-1^ was calculated for the initial and final states, respectively. The DOPG system depicted a D_L_ of 0.012 (± 0.010) x 10^−5^ cm^2^ s^-1^ and 0.014 (± 0.000) x 10^−5^ cm^2^ s^-1^ for the initial and final conformers, respectively. The significant decrease and negligible change of D_L_ in the POPC and DOPG systems, respectively, indicated a differential BMAP27 membrane absorption and binding mechanism (Fig 2A and 2B).

**Table 2:** Average number of hydrogen bonds between membrane lipids and BMAP27 residues.

Bilayer system	R2	R5	R7	K8	K9	K11	K15	K16	H25
POPC	1.11	2.79	0.03	0.07	0.36	0.00	0.03	0.87	0.33
DOPG	3.16	2.62	1.70	1.70	2.20	0.78	1.21	0.77	0.54
TLM	3.32	3.20	0.23	0.23	2.86	1.18	2.39	0.37	0.08
LLM	2.42	3.47	1.05	2.15	1.64	1.14	0.82	2.31	0.54
DOPG-BMAP_mut_	2.17	1.08	2.72	0.59	0.82	0.01	0.54	0.77	0.54
LLM-BMAP_mut_	1.46	1.05	1.21	0.00	0.39	0.00	0.15	0.71	0.29

#### Interaction of BMAP27 with TLM and LLM

**Fig 3:**
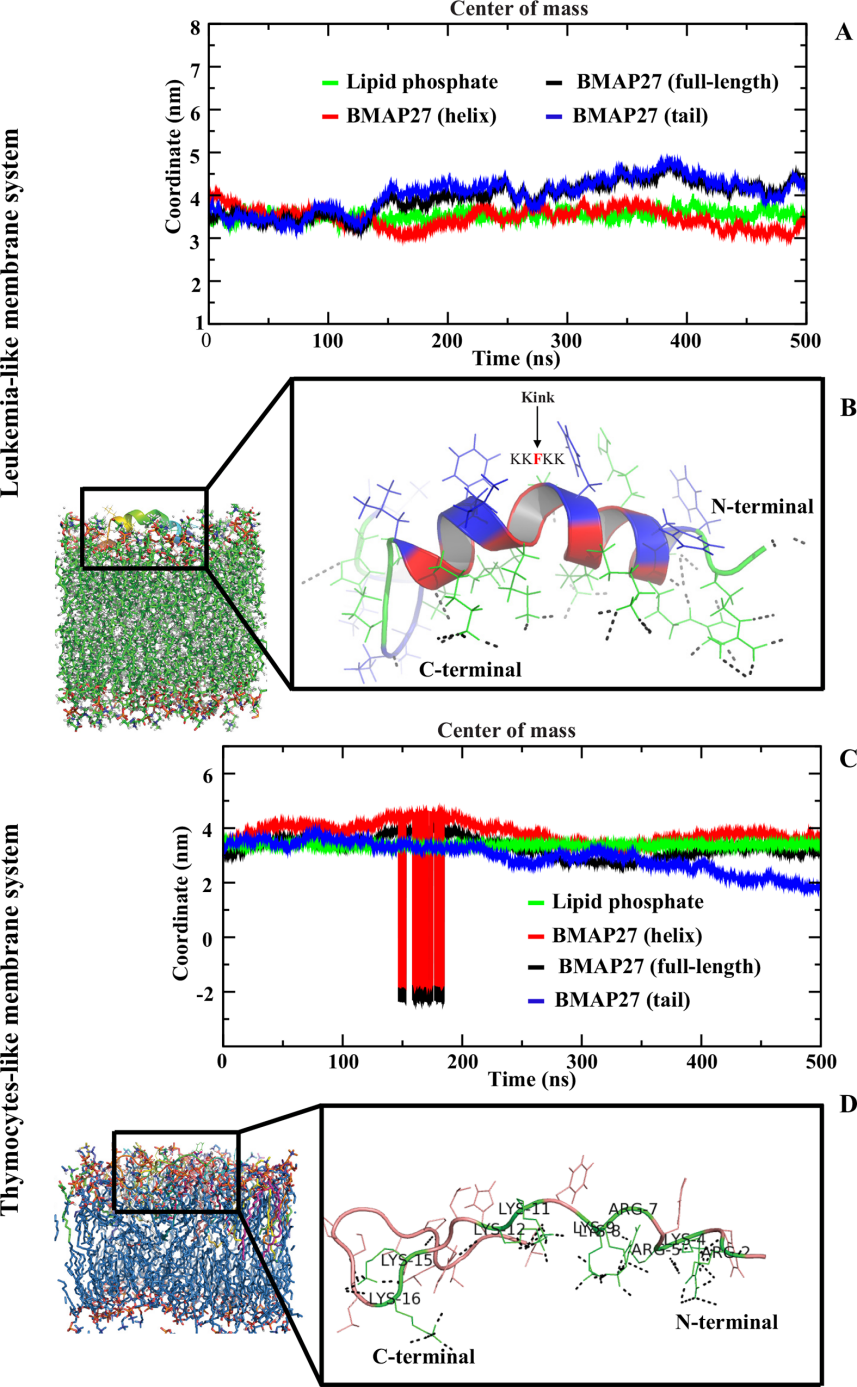
Interaction of BMAP27 with TLM and LLM systems in all-atom MD simulation. (A) The graph illustrates the center of mass of BMAP27 (full-length), helix (3–16), C-terminal residues (17–26) and lipid phosphate atoms. (B) Illustration of interaction of BMAP27 with the LLM. The peptide and lipids are shown as a cartoon and line in PyMOL. The hydrogen bonds between BMAP27 and lipid molecules are shown in black dotted lines. The hydrophobic residues and their solvent exposed side chains are shown in blue. The crucial kink position at F10 is shown as bold and red. (C) Center of mass analysis of BMAP27 in the TLM system. (D) Conformational alternation of BMAP27 and its interaction with the TLM model.

During 0.5 μs MD simulation, a loss of BMAP27 helix conformation was revealed in the TLM system. In contrast, a bent and restrained helical conformation with the central kink at F10 residue was observed in the LLM system (Fig 3). The helix content of BMAP27 was calculated to be > 60% and < 20% in the simulations for the LLM and TLM models, respectively (S2B Fig). The results closely resemble the CD spectroscopy findings of its homologous protein in human that shows partial binding and ~10% helix in mammalian-like membranes as compared to bacterial-like membranes [38]. The number of intermolecular H-bonds were the highest for the helical cationic residues bonding with the lipid phosphate and carboxyl atoms in the TLM and LLM systems, respectively (Table 2). The interaction networks of different lipid components were measured and interpreted during the 0.5 μs simulation. In the TLM system, the highest number of H-bonds was yielded by the PC (average ~14.35) followed by PE lipids (average ~3.14). Relatively strong peptide-lipid bonding network was revealed for the anionic PS (average ~16.20) followed by PC lipids (average ~5.81) in the LLM system. In both membrane models a very weak peptide-cholesterol interaction was calculated, which indicated a limited peptide penetration during the 0.5 μs simulation (S3 Fig). The peptide COM in these two systems exhibited its membrane surface distribution and penetration. The small BMAP27 insertion in the LLM membrane was characterized by its helix penetration (up to ~ 3–5 Å) at the last 100 ns MD simulation (Fig 3A). By contrast, the extended peptide was localized on the TLM membrane surface with a maximum penetration of < 2–3 Å during the ~ 300–350 ns time period (Fig 3C). The peptide C-terminal and helix penetrations were maximum in the TLM and LLM models, respectively, and pointed a distinguish mode of transition. A sharp kink at the center of the helix (F10) in the LLM model led to a peptide reorientation and oblique attachment to the membrane surface. The membrane mediated induced kink in BMAP27 indicated its potential role in providing a suitable orientation for partial peptide penetration and a defined amphipathic environment for strong binding (Table 2) at the membrane interface. Unlike in the POPC system, in the TLM, we observed the unfolded BMAP27 molecule lying on the surface of the bilayer (Fig 3B and 3D). The extended peptide conformation in the TLM with average backbone root mean square deviation (RMSD) of 5.1 Å (cf. average RMSD of 3.5 Å in the LLM) depicted a relatively large peptide translation during the 0.5 μs simulation (S2C Fig). The average D_L_ in TLM was 0.005 (± 0.001) x 10^−5^ cm^2^ s^-1^ and 0.001 (± 0.001) x 10^−5^ cm^2^ s^-1^ for the initial and final conformers, respectively. As in the DOPG system, a comparatively small change with D_L_ of 0.009 (± 0.006) x 10^−5^ cm^2^ s^-1^ and 0.007 (± 0.002) x 10^−5^ cm^2^ s^-1^ was calculated in the LLM systems. This dynamical property corresponded to a strong absorbability of unfolded BMAP27 by the TLM, and weak binding of the kinked helix in LLM system. The analysis for the peptide solvent accessible surface area (ΔG_solv_) showed a small change with average ΔG_solv_ of 126.0 and 136.4 nm^2^ in the TLM and LLM systems, respectively.

The MD simulation of BMAP27_mut_ showed a restrained helical register during the 250 ns time period in both DOPG and LLM systems. In DOPG system, the BMAP27_mut_ molecule was attached parallel to the membrane surface and no peptide tilt was observed. The hydrophobic end was away from the membrane surface, however, no helical loss was observed. On the other hand, in LLM, the hydrophobic C-terminal end of BMAP27_mut_ molecule was partially introduced into the membrane and the helix was placed away from the membrane surface as compared to the wild type BMAP27 (S4 Fig). The average number of H-bonds between the peptide cationic residues and membrane was found to be decreased in both anionic systems in comparison to wild type (Table 2). The loss or significant reduction in the average number of H-bonds between K8/K9/K11 and lipid molecules in LLM system indicated the potential role of the central helix region for the peptide binding and reorientation (Table 2). This region also showed a reduction in the average number of H-bonds in DOPG system. The binding orientation and loss of potential hydrogen bonds indicated a less energetically favorable penetration of BMAP27_mut_ and has been quantified using PMF profile analysis.

### Membrane dependent BMAP27 aggregation

**Fig 4:**
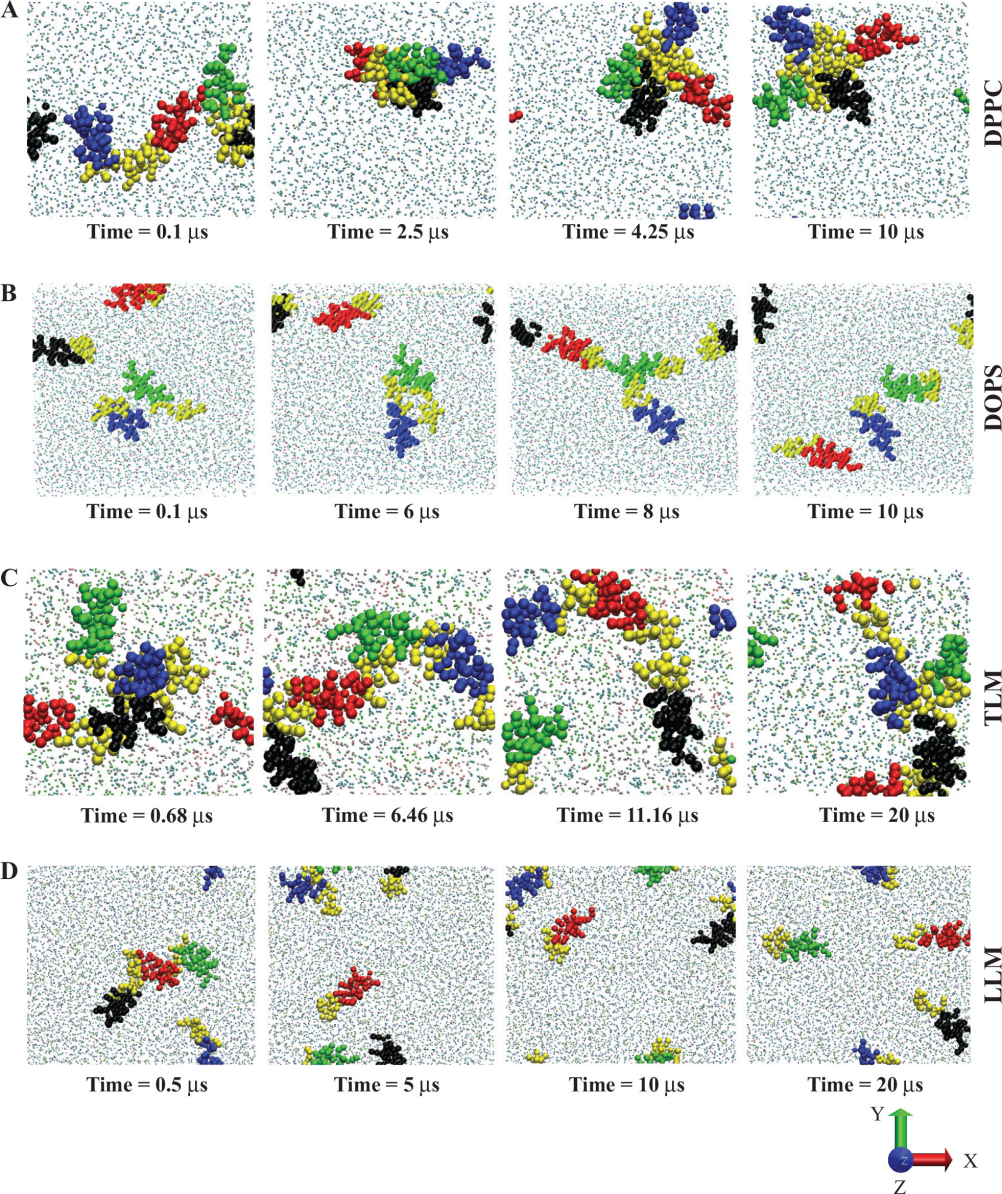
Coarse-grained MD models illustrating interaction between BMAP27 and lipid-bilayers. Snapshots of BMAP27 interacting with (A) DPPC model membrane during 10 μs time period, (B) DOPG model membrane during 10 μs time period, (C) TLM model membrane during 20 μs time period, and (D) LLM model membrane during 20 μs time period. The graphics are generated using VMD program. The α-helices of four different BMAP27 molecules are shown as blue, black, red and green in VDW format and their C-terminal hydrophobic residues as yellow. The lipid molecule types are represented in CPK format.

Here, we investigated the possibility of BMAP27 aggregation and oligomerization at a higher concentration and their effect on the membrane structure in a multi-μs time scale. Interaction in the 1,2-dipalmitoyl-sn-glycero-3-phosphocholine (DPPC) system with P/L of 4:226 presented a stable oligomerization during the 10 μs simulation period. The distantly placed BMAP27 molecules in the initial state formed a micelle-like conformation (S1 File) with the C-terminal hydrophobic residues at the kernel (tetramer formation after ~ 0.4–0.5 μs). The N-terminal cationic helices were placed outward and away from each other due to the electrostatic repulsion and defined a stable and well organized micelle-like conformation (after ~1 μs). The BMAP27 monomers initially formed selective dimers (at ~ 200 ns, and ~ 400 ns) which latter interacted with the membrane surface to constitute a stable tetramer (Fig 4A). On the other hand, a distinguished peptide distribution map was calculated for the DOPG and DOPS systems at variable lipid concentration. Interestingly, in the both systems, we did not observe a stable tetramer and hydrophobic packing. In the DOPS system, peptide homo-dimerization was observed during the first 104 ns period followed by rapid segregation (~188 ns). A relatively unstable oligomerization (Fig 4B) and monomeric interaction of BMAP27 were depicted at the end of the simulation in the both anionic systems (S1 File).

Aggregation of BMAP27 in the DPPC and TLM systems was prominently mediated by the hydrophobic residues at the C-terminus. In addition, involvement of a few helical residues (13–16) for BMAP27 aggregation was identified. Interestingly, algorithm based prediction derived from in vivo experiments using aggregation-propensity scale for natural amino acids (see Materials and Methods) identified these two regions (11–16 and 23–27) as the hot spots of BMAP27 aggregation. A simple MD simulation of four BMAP27 molecules (Table 1) in aqueous solution also revealed the involvement of these hot spot residues in peptide self-assembly. To cross-check this, we further studied the truncated BMAP27 (trBMAP27^1-18^) interaction in the DPPC and DOPS systems. Results showed distinct monomeric interaction of trBMAP27^1-18^ in the DOPS system as seen in the wild-type MD calculation. Interestingly, weak membrane binding and strong aggregation of trBMAP27^1-18^ mediated by the only hot spot region (residues 11–16) was identified in the aqueous phase in the DPPC model membrane (S5 Fig). Although, self-assembly was observed for both BMAP27 and trBMAP27^1-18^ in the DPPC system, the trBMAP27^1-18^ showed significantly weak membrane binding and aqueous phase distribution, which indicated its membrane non-specificity and cytotoxicity.

Unlike in the zwitterionic system, a tetramer with end-to-end (linear) arrangement after ~ 1 μs was identified in the TLM system. The peptides initially aggregated in the solvent and slowly reoriented upon membrane interaction and formed an end-to-end oligomer facing their C-terminals towards the membrane core (Fig 4C). On the contrary, as found in DOPS/DOPG systems, BMAP27 exhibited an unstable oligomer and anionic lipid aggregation in the LLM and hyPLM systems (Fig 4D). A low peptide penetration (as compared to LLM), but selective anionic lipid patches around the BMAP27 molecule was observed in the hyPLM system (S6 Fig). The discriminating lipid aggregation, small membrane intrusion, thickness alteration and shallow angle insertion of BMAP27 in the LLM and hyPLM (S6 Fig) resembled a possible transmembrane mode penetration (toroidal pore like) as proposed for a few other α-helical AMPs [46, 47]. By contrast, the end-to-end oligomerization and tight surface binding in TLM (Fig 4) suggested a different binding and penetration mode which resembles the carpet model mechanism (S2 File).

**Fig 5:**
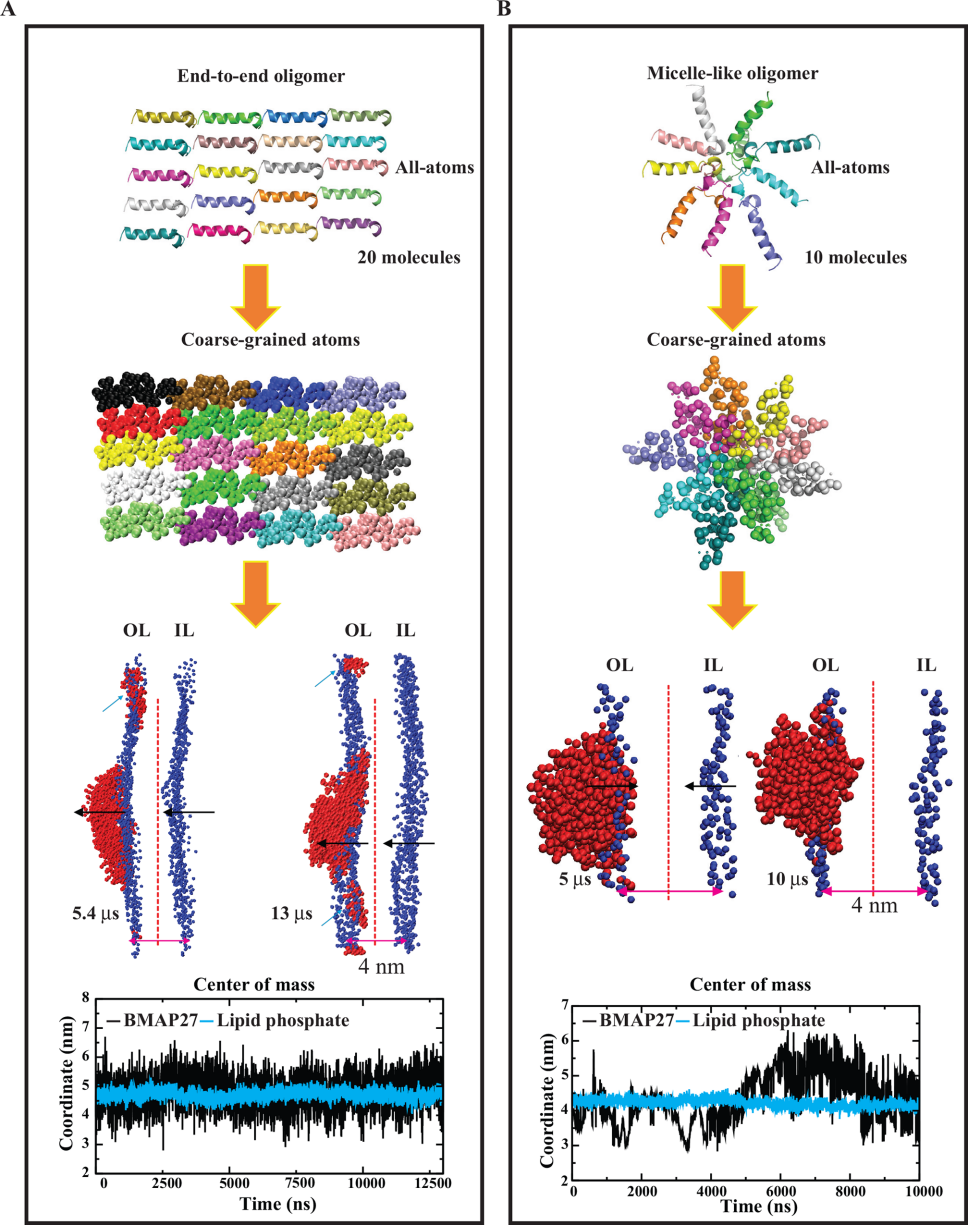
Representation of end-to-end and micelle-like BMAP27 oligomer interaction. (A) End-to-end arrangement, and (B) micelle-like arrangement. The all-atom and coarse-grained models of peptide oligomers are shown as a cartoon and spheres, respectively, and are colored by chains. The relative peptide penetration is calculated from its center of mass to the outer leaflet lipid phosphate atoms as a function of time. The peptide and lipid phosphate atoms are shown in red and blue VDW formats, respectively, in VMD program. Vertical dotted lines divide both leaflets symmetrically and the black arrows indicate the membrane groove formation upon peptide binding. The abbreviations OL and IL denote outer-leaflet and inner-leaflets, respectively. The cyan arrows indicate monomer formation in the end-to-end model system.

The peptide oligomerization, peptide-membrane interaction and membrane disruption mechanisms were highly modulated by the membrane composition and P/L ratio. Thus, high order artificial oligomers were designed considering the micelle and linearly arranged end-to-end conformation (Table 1). Interaction analysis of both oligomers showed a minimal peptide effect on membrane structure and dynamics. Small membrane groove formation and peptide-membrane attachment were identified (Fig 5). However, no spontaneous pore formation or membrane disruption was noticed during the simulation even at high P/L (1:25). Interestingly, slow disaggregation of 3 peptides (out of 20) was identified during the 13 μs period in the linearly arranged system in the anionic membrane environment, indicating further disaggregation over time (Fig 5A). The end-to-end oligomer model presented an average peptide COM of 4.64 ± 0.24 (S.D.) nm with respect to phosphate COM of 4.54 ± 0.08 (S.D.) nm and revealed a surficial distribution at moderate concentration. The micelle-like oligomer exhibited a comparatively high penetration with peptide and phosphate COM of 4.39 ± 0.73 (S.D.) and 4.20 ± 0.12 (S.D.), respectively, at a high concentration (Fig 5B). This comparative analysis suggested that under the substantial peptide concentration and the membrane surface charge, these peptides cause a bilayer deformation by either tight peptide oligomerization (TLM) or selective lipid-molecule aggregation (LLM), which can turgor the cell followed by membrane disruption on a longer time scale. The D_L_ value as listed in S1 Table showed a significant peptide effect on lipid movement for all homogenous membranes and micelle-like oligomers only at high peptide concentration.

#### Effect of BMAP27 on the membrane biophysics

**Fig 6:**
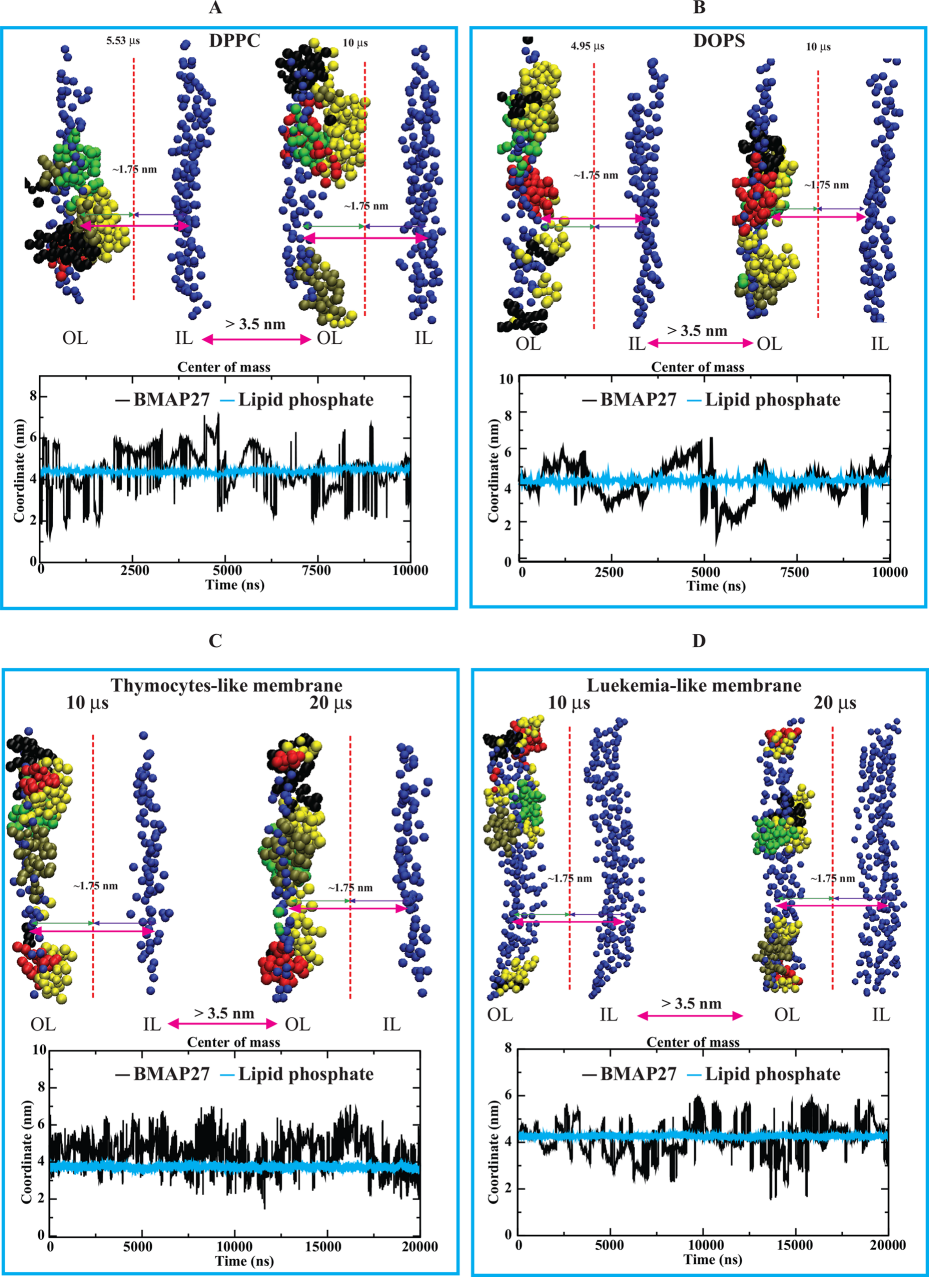
Illustration of BMAP27 interaction and penetration during the multi-μs coarse-grained MD simulation. BMAP27 interaction in (A) DPPC, (B) DOPS, (C) TLM, and (D) LLM model systems. The peptide monomers are colored by chain and shown as tan, black, red and green, and the C-terminal residues as yellow in VDW format in VMD program. The outer and inner leaflets of each bilayer system are denoted as OL and IL, respectively. The vertical dotted line drawn indicates the symmetric division of the total distance between the OL and IL. The center of mass graphs representing the peptide penetration is shown with respect to the lipid head atoms for individual systems and are presented below their corresponding molecular structures.

The peptide penetration was measured by comparing its COM with the COM of lipid head phosphates. In the zwitterionic system, the average distance of BMAP27 peptides from the COM of lipid heads was in the range of 4.44 ± 1.07 (S.D.) nm [4.12 ± 0.90 (S.D.) nm during the period 7–10 μs] with respect to the phosphate COM of 4.41 ± 0.10 (S.D.) nm (Fig 6A). The anionic system presented a relatively high penetration with an average peptide COM of 4.32 ± 1.18 (S.D.) nm [3.14 ± 0.70 (S.D.) nm during 5 to 5.3 μs] with respect to lipid heads 4.25 ± 0.09 (S.D.) nm (Fig 6A and 6B). In the LLM system, the average peptide and phosphate COM were 4.70 ± 1.82 (S.D.) nm and 4.26 ± 0.09 [2.85 ± 1.82 (S.D.) nm during 5 μs to 7 μs], respectively. The hyPLM system presented an average peptide and phosphate COM of 4.22 ± 1.51 and 4.18 ± 0.10 (S.D.) nm, respectively. The TLM model analysis also yielded a higher peptide absorption with average COM of 4.54 ± 0.92 and 3.73 ± 0.11 nm for the peptide and lipid phosphate, respectively (Fig 6C and 6D). Overall, a peptide penetration up to ~ 1.5 nm was calculated for these model systems during the multi-μs period.

**Fig 7:**
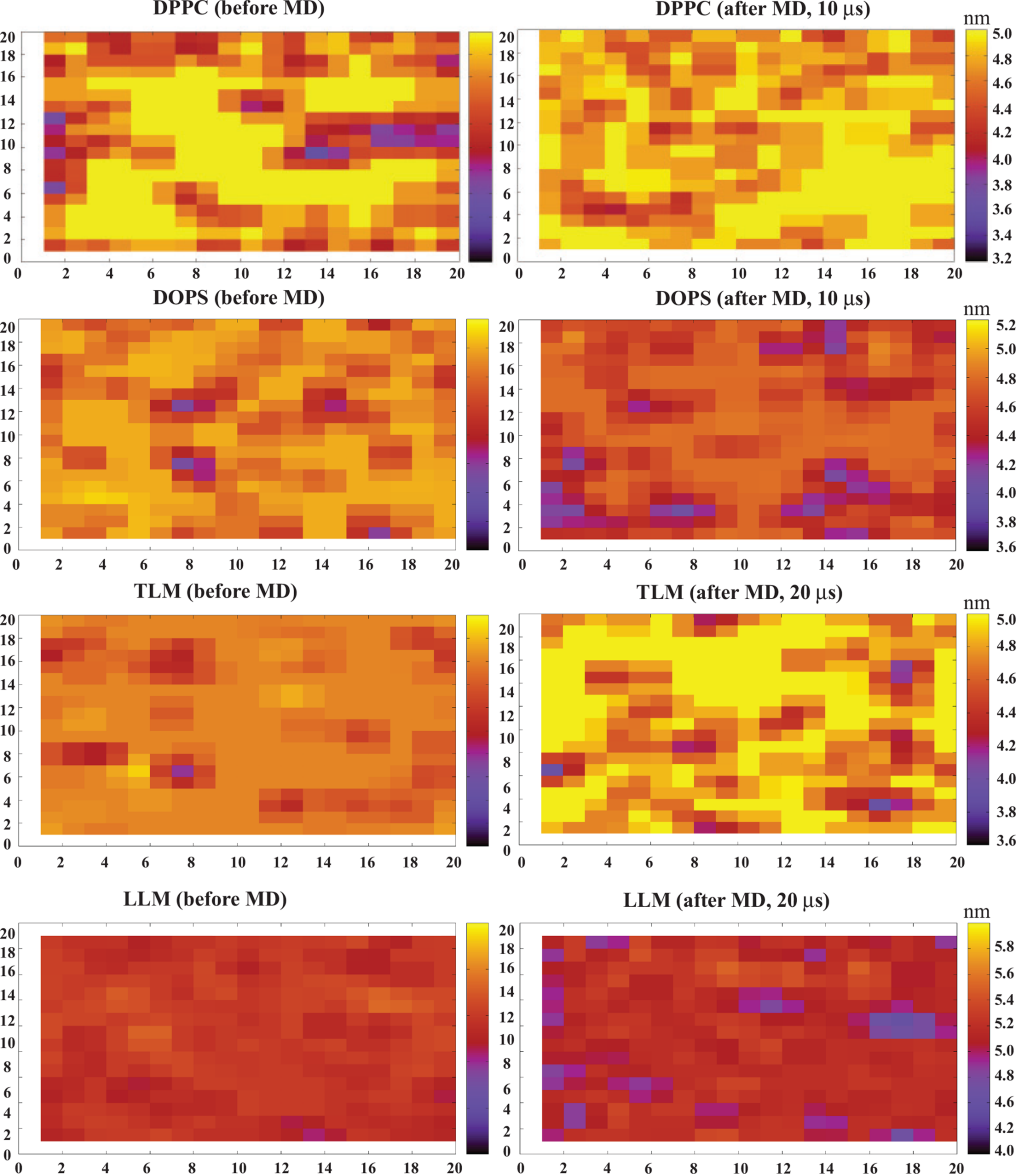
Measurement of bilayer thickness in coarse-grained systems using GridMAT-MD program. The graphics were generated by gnuplot 4.6 using a 20 x 20 matrix distribution. The thickness change monitored through the different colors in the graph are correlated with the distance scale (nanometer) given on the right-hand side of the individual systems. For each system two graphs are shown representing the bilayer thickness before MD (in absence of peptide) and after MD (with peptide) simulation.

Model lipid-bilayer thickness analysis (see Materials and Methods) showed small changes in DPPC bilayer thickness after MD simulation as compared to the DOPS system. Similarly, a significant decrease in the bilayer thickness was calculated for the LLM in comparison to the TLM system (Fig 7). In the DOPS and LLM systems, the changes were relatively large and remarkably distinguishable (in a range from 3.6 to 4.6 nm). The BMAP27 oligomerization on membrane surface and small membrane penetration in zwitterionic and TLM systems suggested requirement of higher peptide density to trigger membrane permeabilization/disruption. This correlates with a possible carpet-like mechanism where the membrane disrupts at relatively high peptide concentrations distributing over the membrane surface. On the other hand, unstable oligomerization, parallel membrane attachment followed by shallow angle penetration and membrane thinning in the anionic and LLM models suggested a mechanism that resembles a toroidal pore mechanism of action.

### Free energy profile analysis

The computational study estimated the PMFs for the BMAP27 molecule as a function of its position perpendicular to the bilayer surface (Fig 2C). The BMAP27 molecule was found to be kinetically trapped at the water/membrane interface followed by reorientation. The peptide bound to the membrane surface in a parallel fashion and tilted during penetration aligning its hydrophobic and hydrophilic regions to the membrane core and polar surface, respectively. At the membrane interface (z ~ 2.5 to 1.5 nm) an apparent average free energy (ΔG) of ~ -4.96 ± 1.30 kcal mol^-1^ was estimated for the anionic (DOPG) system. In comparison, the neutral (POPC) membrane depicted an average ΔG of ~ -0.90 ± 0.13 kcal mol^-1^. The BMAP27 molecule rendered a positive and negative ΔG of ~ +0.17 ± 0.83 and -4.58 ± 1.30 kcal mol^-1^ in the TLM and LLM systems, respectively. As shown in Fig 2C, the membrane surface interaction kinetics indicated the selectivity of BMAP27 to the anionic systems. The average ΔG estimation from the PMF profile at the lipid hydrophobic core (z ~ -1.5 to 1.5) presented -2.88 ± 0.71; 0.73 ± 0.05; 1.49 ± 0.65 and -2.85 ± 0.96 kcal mol^-1^ for the DOPG, POPC, TLM and LLM systems, respectively. The comparative PMF analysis showed a relatively high energy barrier at the membrane surface for the neutral systems. As seen from the height of energy barrier (Fig 2C), positive energy peaks appeared in POPC and TLM systems during the peptide transition from the water phase to the water/membrane interface. At the exofacial membrane surface, this energy barrier was slightly reduced in POPC system and presented an energy minimum of -2.0 kcal mol^-1^. But, the height of the average free energy barrier remains positive for the TLM system with energy minimum of -0.67 kcal mol^-1^. This relatively high energy barrier could be due to the membrane rigidity contributed by the substantial molar concentration of cholesterol. The PMF minima for the anionic systems with respect to the water phase were ~ 5 times larger than the POPC/TLM systems. The comparative analysis of PMF profiles showed a favorable peptide insertion at the water/membrane interface; however, at the bilayer core region the permeation was less energetically favorable with energy maximum of ~ -3.5 kcal mol^-1^. The umbrella sampling based PMF profile calculations in BMAP27 estimated a binding energy that is consistent with the experimental and computational results obtained in other α-helical antimicrobial peptides [48]. The PMF analysis in the BMAP27_mut_ showed a small ΔG change in DOPG system with an average value of -4.82 ± 1.25 and -3.17 ± 1.13 kcal mol^-1^ at the exofacial and hydrophobic lipid core regions, respectively. The LLM system on the other hand depicted a substantial change in the free energy profile of BMAP27_mut_ and indicated the onset of a large surface penetration energy barrier for the peptide molecule (Fig 2C). The average ΔG of the BMAP27_mut_ in LLM system was calculated to be -2.45 ± 0.44 and -0.84 ± 0.41 kcal mol^-1^ for the bilayer surface and core, respectively. The substantial PMF and hydrogen bond alteration (Table 2) in BMAP27_mut_ indicated its low affinity to permeabilize the membrane surface and suggested a possible loss of function.

## Discussion

Atomistic simulations of AMPs in model bacterial membrane systems have been widely studied in recent years. These studies consider homogenous or heterogeneous lipid systems [13, 14, 49–51]. However, the mechanism of anticancer activity of some selected cell-penetrating peptides including cathelicidins in cancer mimicking lipid systems have been poorly explored. The major difficulties for such atomistic studies depend on the lipid selectivity, peptide-lipid ratio, simulation time length, force field standardization etc. Considering these, here we studied the BMAP27 interaction and its effect on homogenous (anionic and zwitterionic) and non-homogeneous (TLM, LLM and hyPLM) model systems with different lipid compositions (Table 1).

Our data from this study shed light on the environment dependent and force field independent behavior of BMAP27 which can be correlated with its cytotoxicity and the mechanism of cell-disruption [13]. The MD simulation in aqueous solution revealed an unfolded conformation in the absence of hydrophobic environment. The all-atom MD analysis in the anionic, zwitterionic, TLM and LLM systems showed that BMAP27 attraction and initial membrane orientation were mediated through the potential hydrogen bond formation between its cationic residues and lipid heads (Table 2). The inequality of electrostatic and hydrophobic forces in peptide-lipid interfaces further reoriented the peptide molecule in a membrane selective fashion.

The secondary structural integrity may also depend on the P/L concentration as seen in human cathelicidin [38]. Despite of the lipid heterogeneity, the intermolecular interaction among peptides in a multimer state could help in restraining the helicity. In our all-atom MD simulation, we focused on the conformational alteration in BMAP27 at a moderate concentration. The results correlated with the folding and unfolding characteristic of its homologous human cathelicidin as a function of the membrane composition and surface charge. Peptide helix stability was favored by electrostatic interaction and aggregation of anionic lipids around the peptide molecule. This arrested the peptide backbone translation (low RMSD) and provided a well-defined amphipathic orientation across the membrane interface for the interaction and penetration. On the contrary, the peptide translational diffusion was faster in the absence of membrane surface charge. The large peptide conformational space (Fig 3) also significantly affected the lipid lateral movement as reveled from their lateral diffusion. The distinguished behavior of BMAP27 depends on the lipid heterogeneity in the chemical composition of lipid molecules which vary in healthy, proliferative, tumor and microbial cells [52, 53]. The net membrane surface charges and the anionic lipids in microbes, tumor and proliferative cells were thought to be the major contributing factors for the cytotoxic activity of such peptides in the presence of poly-cationic substances [2–4]. The restrained folded conformation followed by membrane penetration at shallow angles in the microbial (anionic) and LLM systems indicated a transmembrane mode membrane insertion that mimics the toroidal pore mechanism of cell disruption. On the contrary, the extended BMAP27 conformation and translation (high RMSD) due to low electrostatic interaction in the zwitterionic and TLM systems along with tight surficial absorption suggested a concentration dependent peptide aggregation for membrane disruption (Figs 1 and 2). The membrane dependent BMAP27 folding and unfolding indicated a possible correlation with its cytotoxicity, but it is difficult to quantify at this structural level on a limited time scale of simulation. Previous studies also showed that other cathelicidins, α-helical and β-sheet AMPs oligomerizes with stable secondary structure to mediate their antimicrobial action [54–56]. Hence, the unstructured and structured conformations of BMAP27 in the zwitterionic and anionic lipid systems pointed a possible non-transmembrane mode (oligomer) and transmembrane mode (monomer) cytotoxic actions, respectively.

The helical kink induced at the center (F10) of the BMAP27 in the LLM/water interface facilitated the peptide reorientation for possible transmembrane mode transition. These temporary kinks are crucial for the peptide attachment and optimize the hydrophobic moment (Fig 3) for an ideal membrane binding and penetration as observed in a few other helical AMPs [23, 57, 58]. The kinked conformation in BMAP27 ascertained an inclined orientation with hydrophobic side chains facing polar environment that stimulated BMAP27 penetration via hydrophobic movement for optimal peptide-lipid fusion. The helical reorientation and tilt in BMAP27 resembled other α-helical AMP actions for which the AMP tilt at an angle ~30–60˚ with respect to the bilayer normal as studied by NMR and vibrational spectroscopy [59, 60]. On a longer time scale of MD simulation, a profound peptide tilt (~ 45°) can be ensured as revealed from our biased ^sp^DOPG and umbrella sampling studies. This suggested a possible transmembrane mode membrane transition of BMAP27 in bacterial and tumor cell membrane systems (S2A Fig).

From all-atom MD calculations, we are able to explore the BMAP27 attraction, binding, orientation and effects on membrane structure and dynamics at a moderate P/L concentration. However, our unbiased all-atom MD results for 0.5 μs period are not sufficient to access the spontaneous peptide insertion in any of the model lipid-bilayer systems. In fact, a few recent studies on a time scale of ~1–10 μs also reported only surficial distribution of a few α-helical AMPs in different bilayer systems [46, 50, 51, 61]. Correlating our findings with previous studies, we best understood that the antimicrobial and/or anticancer action depend on the time scale of action and the peptide concentration. Structural interpretation over millisecond to second or even longer time is required to explore the AMP mediated cell lysis mechanism that includes peptide attraction, distribution, aggregation, orientation, penetration and cell-membrane disruption. However, simulations on much longer time scales are currently beyond our computational facility. A moderate time scale (multi-μs) MD simulation can identify all major mechanisms, excluding the cell-disruption as reported in a few other AMPs [46, 50, 61]. The high BMAP27 concentration and long simulation time (10–20 μs) in CG models provided an alternative platform to investigate their membrane binding and disruption mechanism. The fixed BMAP27 structure in our CG-MD simulation limited the structural insights into the BMAP27 secondary conformational alteration as seen from the all-atom MD simulation.

Here, the CG models comprising four peptide molecules highlighted the importance of the C-terminal hydrophobic end (C-ter) for their selective AMP action. The fast attraction and initial parallel attachment of BMAP27 molecules to the bilayer normal correlated to the all-atom MD findings. Moreover, like the all-atom model, CG systems displayed the peptide hydrophobic and amphipathic domains align to the membrane core and polar interface, respectively. The hot spot analysis of BMAP27 aggregation and trBMAP27^1-18^ MD simulation study (S5 Fig) further supported the crucial involvement of the C-ter for the peptide cytotoxic activity in normal cells. The role of peptide hydrophobicity in BMAP27 of their self-assembly has also been explored by confocal microscopy and fluorescence experiments. Alanine substitution of phenylalanine/leucine residues in BMAP27 or truncation of the C-ter showed significant effect on peptide cytotoxicity, aggregation and membrane binding [9, 10, 12]. Cytotoxicity of BMAP27 to healthy cells acts at a relatively high concentration (~ 30 μM) as compared to their antimicrobial activity (~ 1.4 μM). Furthermore, the microbicidal activity remains unchanged upon truncation (trBMAP27^1-18^) of the C-terminus hydrophobic residues. This suggested the cytotoxicity of BMAP27 at a higher concentration in normal cells is mediated by the C-ter residues that forms peptide self-assembly on the membrane surface as seen from the CG-MD studies (Fig 4). Stable oligomerization of BMAP27 in aqueous and DPPC models, distinctly showed engagement of the C-ter forming a micelle or end-to-end aggregation. Truncation of the C-ter suppressed the hydrophobic force mediated peptide adhesion and transition (S5 Fig) which is needed to surmount the positive ΔG barrier in the neutral systems. On the contrary, in the anionic membrane systems, the favorable energy at the membrane interface region allowed an easy peptide penetration. The differential energy barriers for BMAP27 absorption by cell/species specific heterogeneous membranes indicated their selective cytotoxic activity. Moreover, the intermolecular hydrogen bonding and PMF profile analysis of BMAP27_mut_ also showed a less favorable binding kinetics in anionic systems. This result agrees with the experimental findings that showed significant change in BMAP27 permeabilizing efficacy in anionic and zwitterionic membranes upon Leu-Ala or Phe-Ala mutation or net hydrophobicity alteration [12]. Hydrophobic force mediated oligomerization in the zwitterionic system (mutated BMAP27 cannot permeabilize the membrane), and electrostatic force mediated monomeric penetration of BMAP27 in anionic systems (mutated BMAP27 can permeabilize the membrane) strongly correlated to the experimental observations. The steered MD and umbrella sampling displayed the preferential binding mode and orientation of BMAP27 in different membrane systems. The favorable and unfavorable ΔG indicated a spontaneous and non-spontaneous membrane insertion potency of the target BMAP27 molecule in DOPG/LLM and POPC/TLM systems, respectively. The initial peptide attachment at the water/membrane interface is kinetically favorable and provides an oblique orientation in anionic systems. But, the slow peptide reorientation on the membrane surface in zwitterionic systems is kinetically barred which may produce surficial adsorption. The peptide adsorption on membrane surface could also be due to the insufficient sampling and the selected time scale per umbrella. We also observed local membrane deformation and organization due to this strong electrostatic interaction between cationic peptides and anionic lipids (S6 Fig). The CG-MD results for multi-μs time period showed the membrane mediated binding mechanism and permeabilization efficacy (Figs 4 and 5) which can be correlated to the antimicrobial or anticancer activity of BMAP27. Comparative study of TLM, LLM and hyPLM was considered to investigate the peptide arrangement due to asymmetric phospholipid distribution and the effect of peptide on selective phospholipid rearrangement.

Replacement of major anionic lipids with zwitterionic lipids and vice-versa affected the peptide distribution pattern on the membrane surface. In the LLM and hyPLM systems, the peptides were steadily and selectively found at negatively charged rich regions and engendered lipid aggregation as seen in other cationic peptides [46]. In this study, both all-atom and CG-MD findings supported the evidence of selective peptide insertion and attachment to the heterogeneous membrane surface. The strong coulombic forces created a much stronger binding energy and restricted the hydrophobic pull and multimer formation. The anionic lipid aggregation in the LLM and hyPLM suggested selective lipid homogeneity and membrane deformation (S6 Fig). In the TLM system, the peptide aggregation and surface absorption were largely driven by the hydrophobic interactions with small peptide-lipid bonded interaction. The peptide surface aggregation and absorption confined the lipid head translation and intra-molecular transition, and affected the lipid lateral diffusion (S1 Table). The BMAP27 mediated selective lipid aggregation and bilayer reorganization, and/or induction of bilayer rigidity by surface aggregation are the possible mechanism of cell disruption. At higher P/L, these peptides exhibited the formation of a significant lipid bilayer groove and induced bilayer thinning along with a steady rise in ΔG_solv_ around the peptide molecules. The significant changes in the lipid lateral diffusion in high P/L ratio may also be a factor for the concentration dependent cytotoxicity of BMAP27.

## Conclusion

In conclusion, we have explored the peptide attraction, folding, aggregation and kinetics in the anionic, zwitterionic, healthy and tumor-like model membranes by employing unbiased MD simulations longer than 100 μs. The structural findings depicted BMAP27 absorption and insertion on the outer leaflet of the membrane along with lipid reorganization, deformation and displacement. In addition, structural and kinetic studies of BMAP27 mutant highlighted the importance of the central helix attachment and kink formation for the peptide insertion. Unfortunately, under the present computational limit we did not notice any spontaneous BMAP27 insertion or equilibrium pore formation during our multi-microsecond time scale of MD simulation. However, comparative and systematic analysis identified stable oligomerization and linear distribution of BMAP27 (micelle or end-to-end arrangement) on the TLM/zwitterionic membrane surface. This hypothesizes a mechanism resembling the carpet-model like cell membrane disruption. On the contrary, unstable oligomers and monomeric interaction of BMAP27 in the LLM/anionic membrane models with a shallow angle penetration suggested a transmembrane mode membrane insertion that resembles the toroidal pore mechanism of action. Free energy profile analysis suggested the existence of a favorable and an unfavorable energy barrier for the anionic and zwitterionic membrane systems, respectively. This can be correlated to the cell specific selective cytotoxic activity of BMAP27. Using unbiased MD simulation, we best understood that unwrapping the complete antimicrobial and anticancer mechanism of action is beyond the present scope of computational simulation. However, these findings profoundly provide the structural insights into the mechanism of selective BMAP27 cytotoxic action towards healthy and cancer cells. Furthermore, these findings can be referenced to rationally design more potent BMAP27 analogues with higher cell/species specificity.

## Supporting Information

S1 Fig Secondary structure profiling of BMAP27 by VMD. (A) BMAP27 conformation in POPC, (B) in DOPG model membrane systems. The secondary structure color legends are T: turn, E: β-sheets, B: isolated bridge, H: α-helix, G: 3–10 helix, I: Pi-helix, and C: coil. The frame numbers are derived from the compressed trajectory and represent the time scale as a function of 0.5 μs.
(PDF)

S2 Fig Peptide tilts, helicity and backbone stability analysis in different systems. (A) BMAP27 tilt during the 100 ns all-atom MD simulation in a deeply buried DOPG system. The peptide is shown as a cartoon, lipid as blue and water as red lines in VMD. The initial and final BMAP27 orientation is shown in green and red, respectively. (B) The graph illustrates the percentage time of helicity conservation with respect to the simulation time period in TLM and LLM all-atom MD systems, and (C) RMSD graph of the backbone atoms of BMAP27 in TLM and LLM all-atom membrane systems.
(PDF)

S3 Fig Intermolecular hydrogen bond analysis. Graph represents the formation of hydrogen bonds (in numbers) between BMAP27 and major lipid components with respect to MD simulation time periods.
(PDF)

S4 Fig Interaction of BMAP27 mutant with anionic systems. (A) Illustration of BMAP27_mut_ and LLM interaction, (B) BMAP27_mut_ and DOPG lipid-bilayer interaction. The initial and final MD snapshots for BMAP27_mut_ interaction are shown in left and right panel, respectively. The BMAP27_mut_ molecule is shown as cartoon, lipids as green lines and water as red lines in PyMOL.
(PDF)

S5 Fig Illustration of oligomerization of truncated BMAP27 (1–18) in DPPC and DOPS system. The peptide molecules are colored as blue, red, orange and grey, and the predicted hot spots (residue 11–16) for aggregation in AGGRESCAN are shown in yellow. The peptide and lipid molecules are represented in VDW and CPK, respectively, and solvent molecules as red dotted circles in VMD.
(PDF)

S6 Fig Snapshots of the coarse-grained model of peptide-lipid interaction in VMD after 20 μs time period. The peptide molecules are colored as blue, red, green and black, and the lipid molecules as cyan for the three different heterogeneous membrane systems (TLM, LLM and hyPLM). The anionic lipid molecules are shown in cyan, and yellow represents the peptide C-terminal residues. All coarse grained beads are represented in VDW format. Selective aggregations of BMAP27 surrounding anionic lipids are indicated by arrows.
(PDF)

S1 File Molecular interaction of BMAP27 with DPPC and DOPS membrane systems. The N-terminal α-helical regions of four different BMAP27 molecules are shown in black, red, green and blue, and the C-terminal residues (19–27) are shown in yellow. The visualization was generated using the VMD movie maker.
(MP4)

S2 File Molecular interaction of BMAP27 with TLM and LLM membrane systems. The N-terminal α-helical regions of four different BMAP27 molecules are shown in black, red, green and blue, and the C-terminal residues (19–27) are shown in yellow. The anionic lipids (DOPS) in the LLM lipid-bilayer is shown in purple. The visualization was generated using the VMD movie maker.
(MP4)

S1 Table Effect of BMAP27 on the lateral diffusion (D_L_) of lipid molecules in CG-MD models. (DOCX)
