# Supplementary figures and images for: Membrane Mediated Antimicrobial and Antitumor Activity of Cathelicidin 6: Structural Insights from Molecular Dynamics Simulation on Multi-Microsecond Scale
Source: PLoS One. 2016 Jul 8;11(7):e0158702. doi: 10.1371/journal.pone.0158702 (PMC4938549; doi:10.1371/journal.pone.0158702)

A

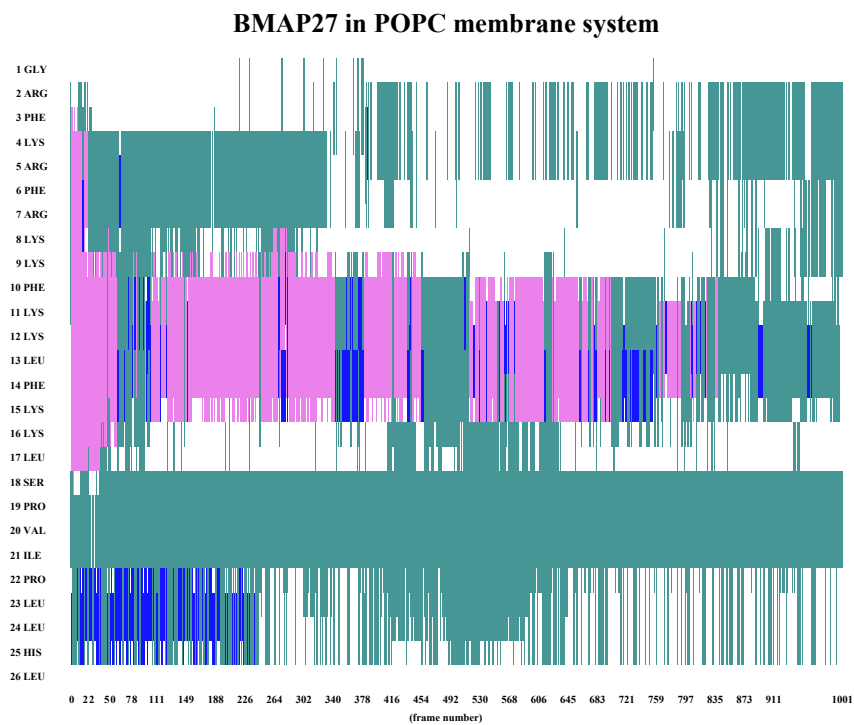

B

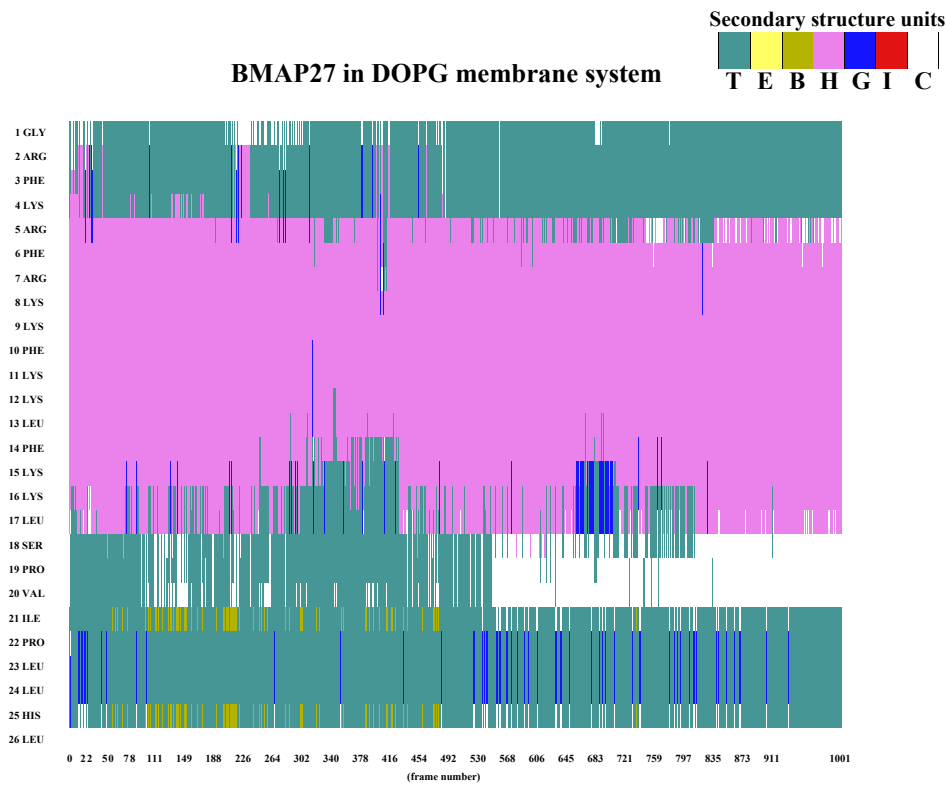

Supplement: S1 Fig — (A) BMAP27 conformation in POPC, (B) in DOPG model membrane systems. The secondary structure color legends are T: turn, E: β-sheets, B: isolated bridge, H: α-helix, G: 3–10 helix, I: Pi-helix, and C: coil. The frame numbers are derived from the compressed trajectory and represent the time scale as a function of 0.5 μs. (PDF) [file pone.0158702.s001.pdf]

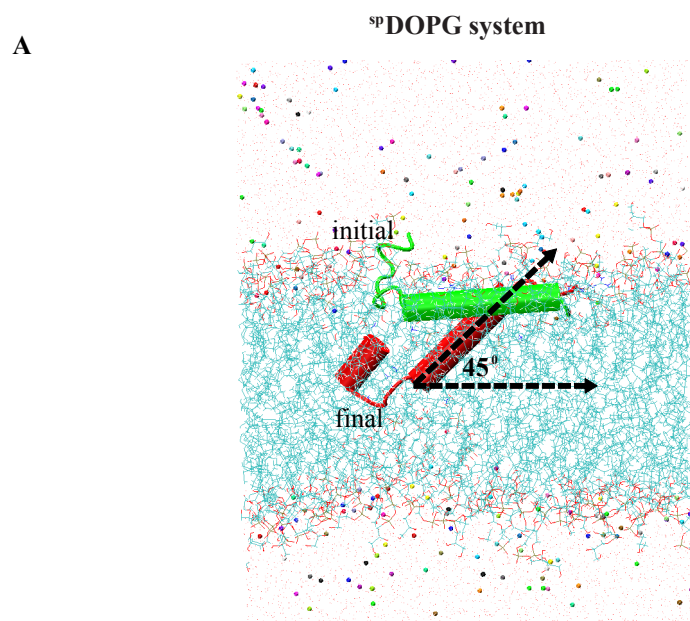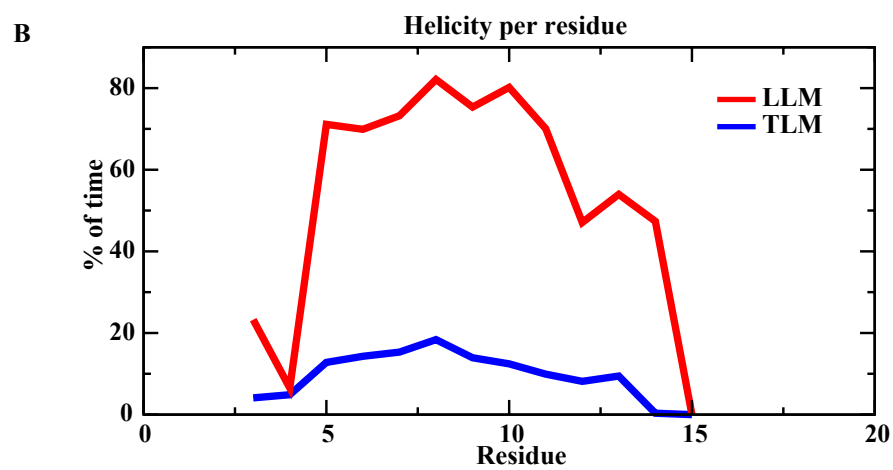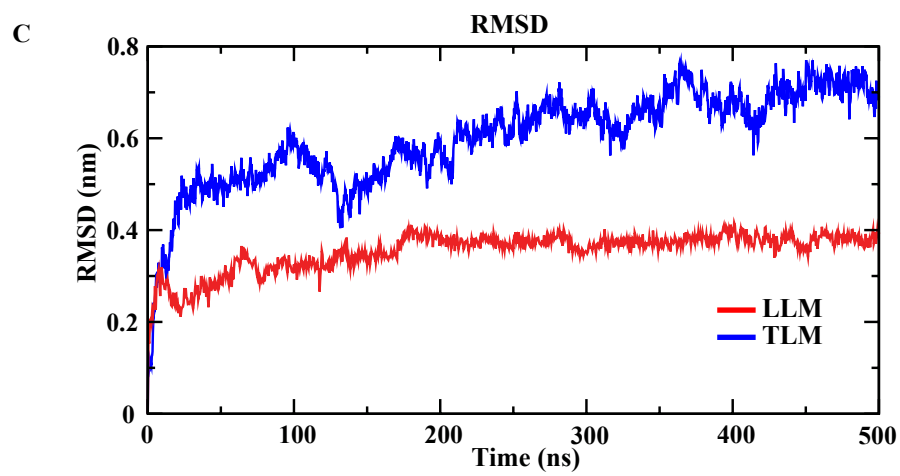

Supplement: S2 Fig — (A) BMAP27 tilt during the 100 ns all-atom MD simulation in a deeply buried DOPG system. The peptide is shown as a cartoon, lipid as blue and water as red lines in VMD. The initial and final BMAP27 orientation is shown in green and red, respectively. (B) The graph illustrates the percentage time of helicity conservation with respect to the simulation time period in TLM and LLM all-atom MD systems, and (C) RMSD graph of the backbone atoms of BMAP27 in TLM and LLM all-atom membrane systems. (PDF) [file pone.0158702.s002.pdf]

A **Thymocytes-like membrane system**

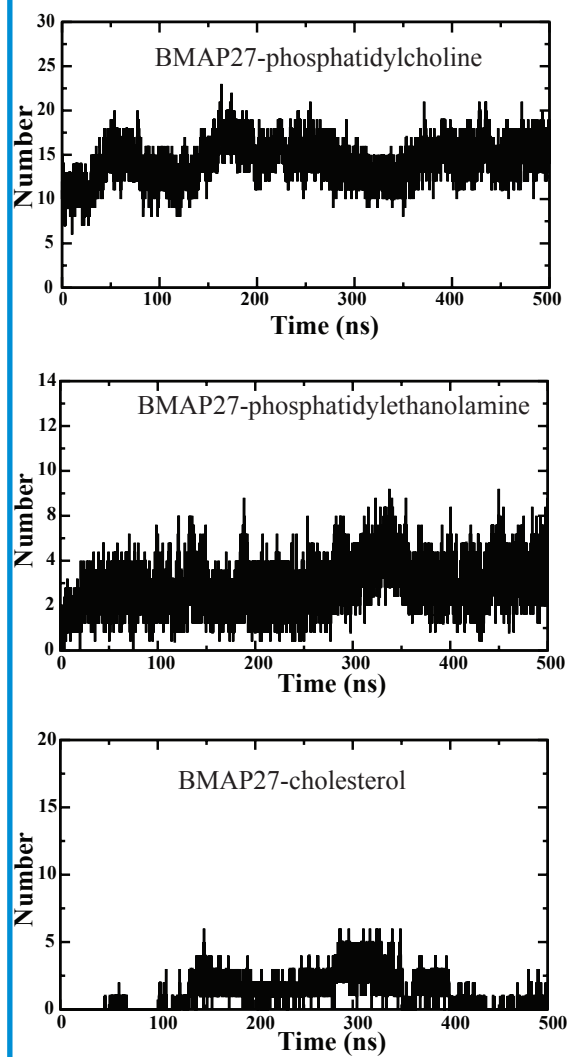

B **Leukemia-like membrane system**

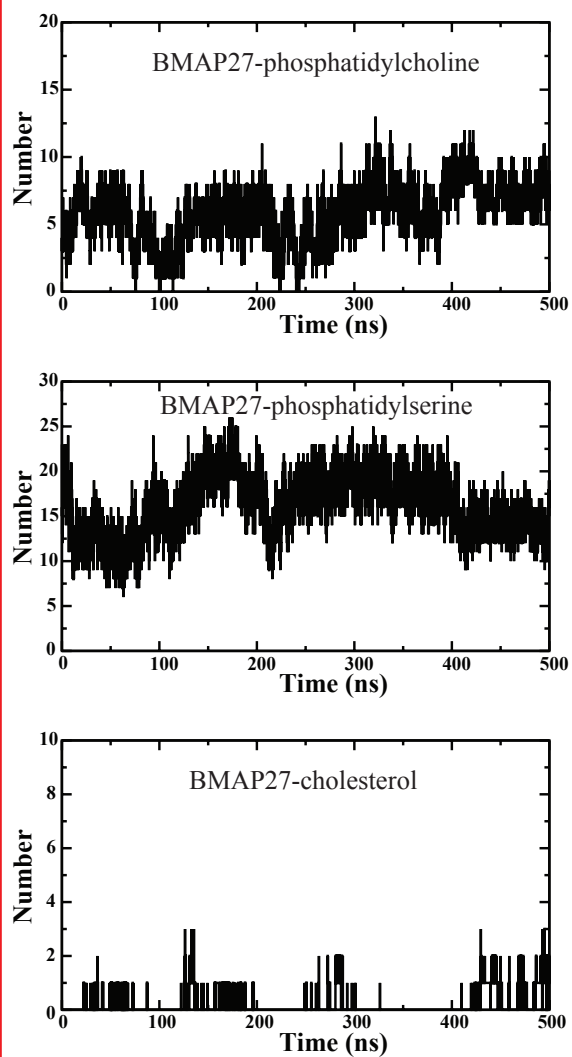

Supplement: S3 Fig — Graph represents the formation of hydrogen bonds (in numbers) between BMAP27 and major lipid components with respect to MD simulation time periods. (PDF) [file pone.0158702.s003.pdf]

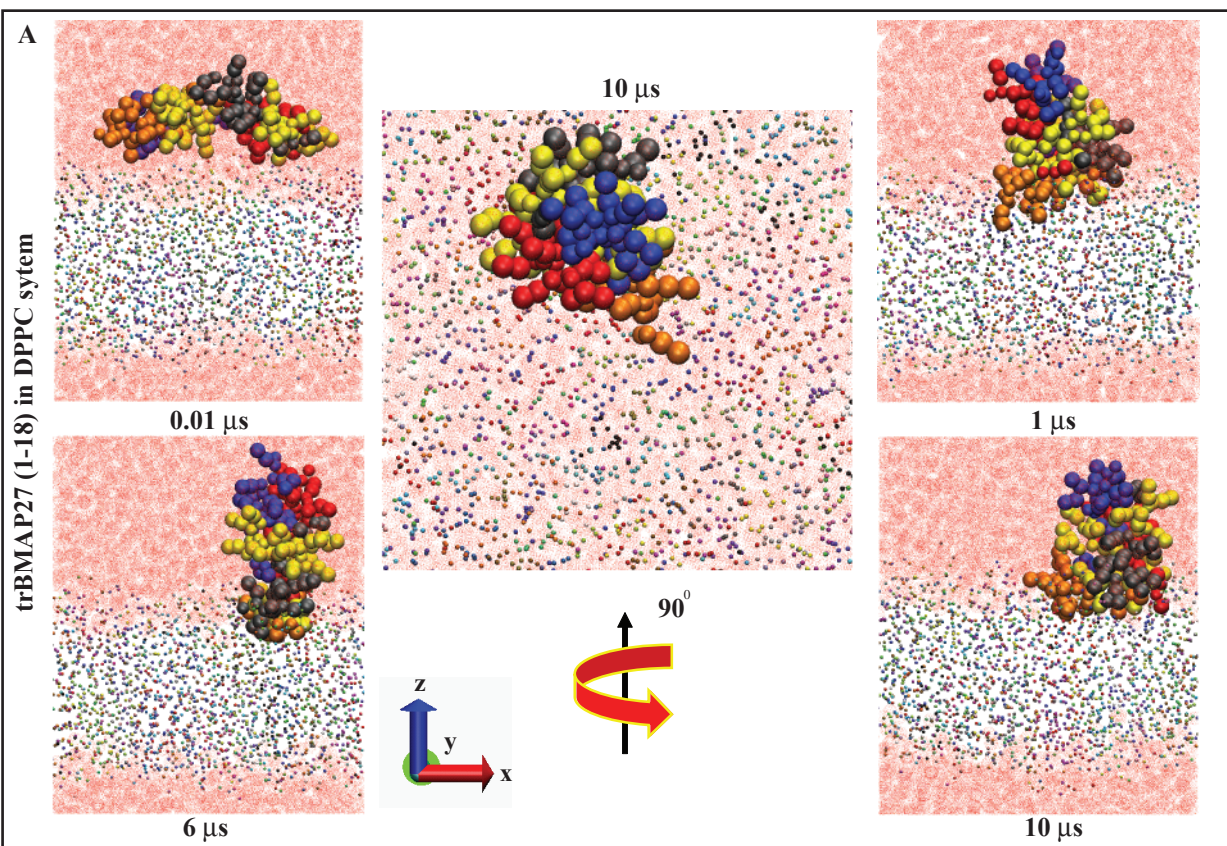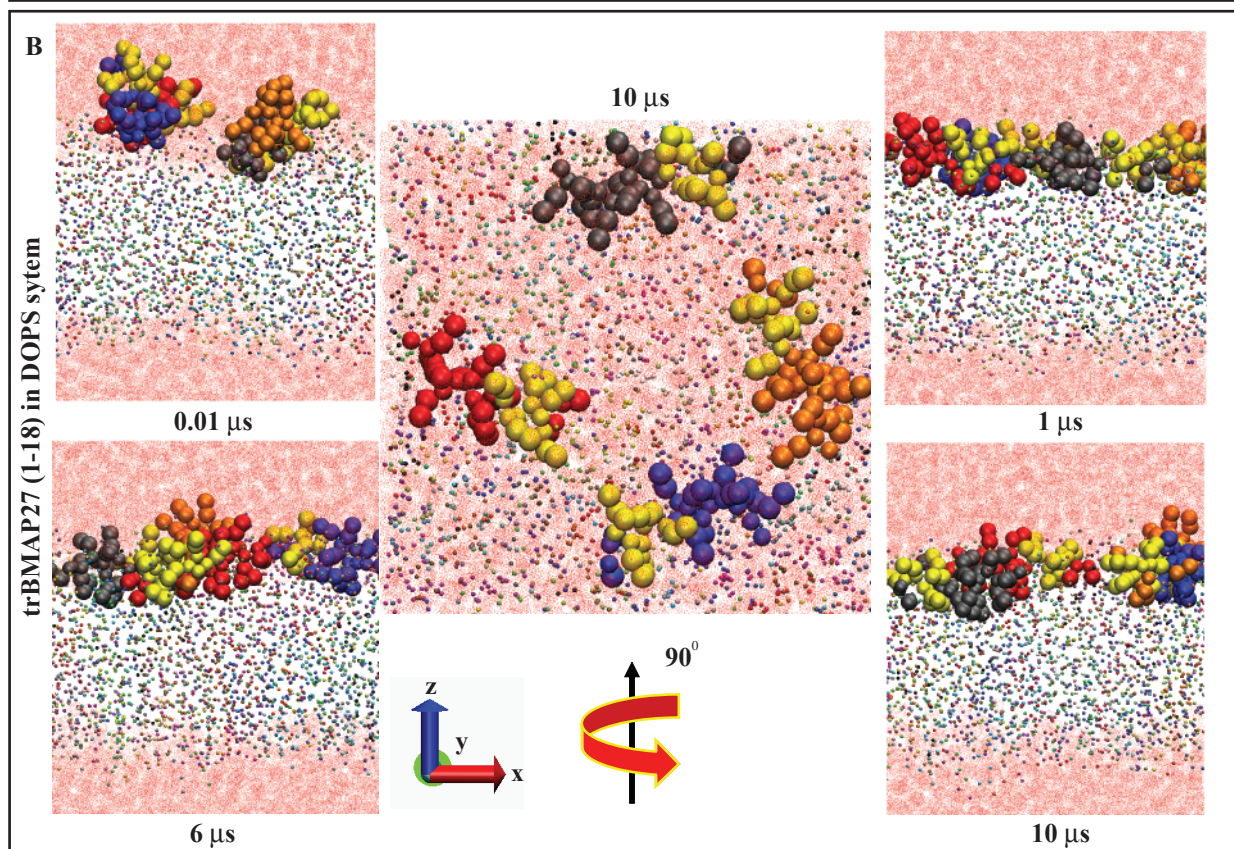

Supplement: S5 Fig — The peptide molecules are colored as blue, red, orange and grey, and the predicted hot spots (residue 11–16) for aggregation in AGGRESCAN are shown in yellow. The peptide and lipid molecules are represented in VDW and CPK, respectively, and solvent molecules as red dotted circles in VMD. (PDF) [file pone.0158702.s005.pdf]
