# Supplementary material for: Membrane Mediated Antimicrobial and Antitumor Activity of Cathelicidin 6: Structural Insights from Molecular Dynamics Simulation on Multi-Microsecond Scale
Source: PLoS One. 2016 Jul 8;11(7):e0158702. doi: 10.1371/journal.pone.0158702 (PMC4938549; doi:10.1371/journal.pone.0158702)
Supplement: S4 Fig — (A) Illustration of BMAP27mut and LLM interaction, (B) BMAP27mut and DOPG lipid-bilayer interaction. The initial and final MD snapshots for BMAP27mut interaction are shown in left and right panel, respectively. The BMAP27mut molecule is shown as cartoon, lipids as green lines and water as red lines in PyMOL. (PDF) [file pone.0158702.s004.pdf]

$BMAP27_{mut}$ interaction with TLM system

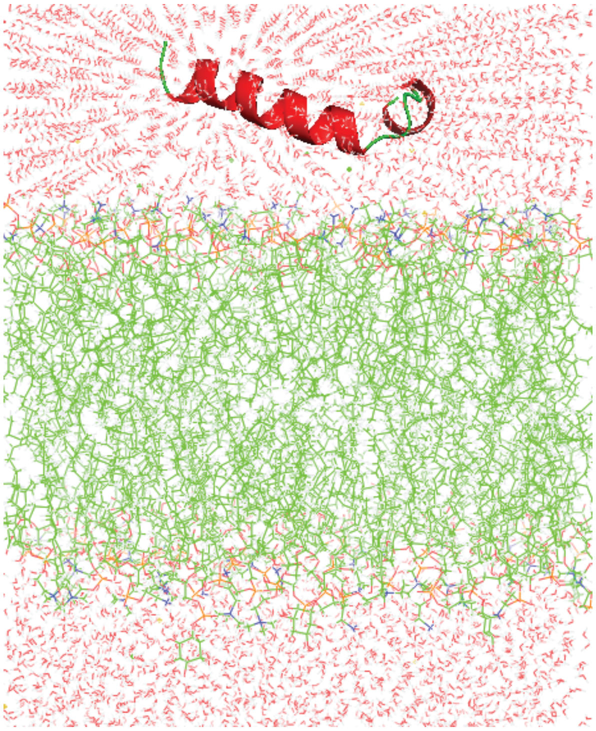

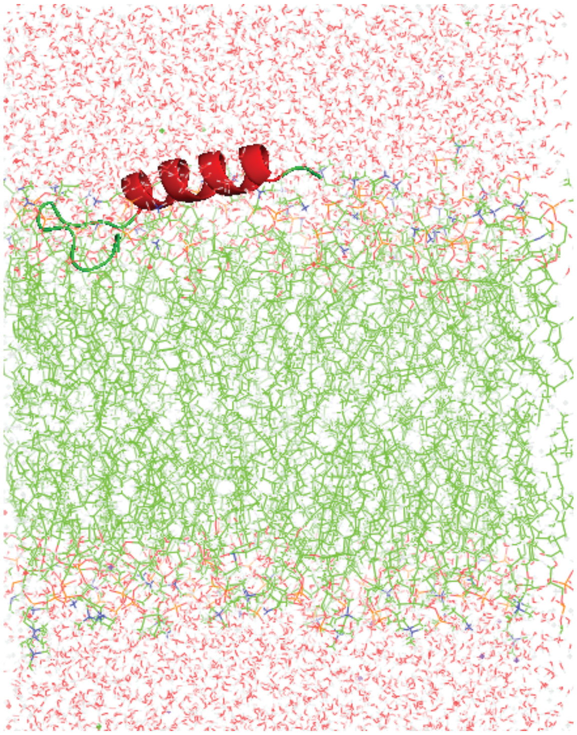

$BMAP27_{mut}$ interaction with DOPG system

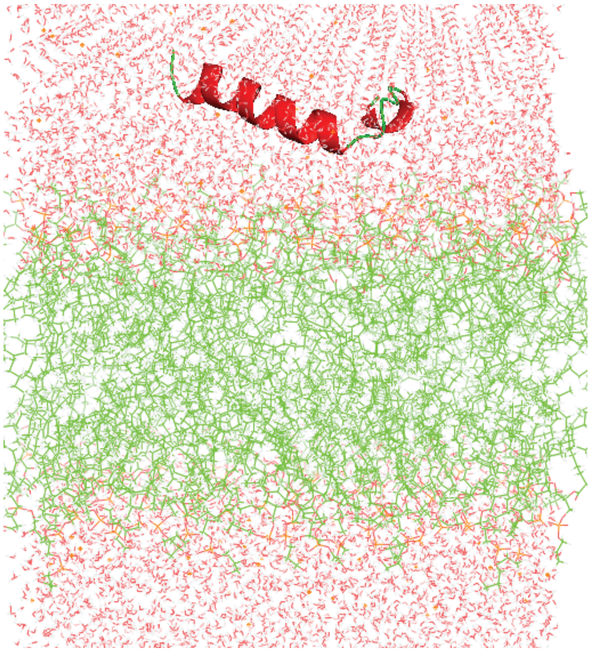

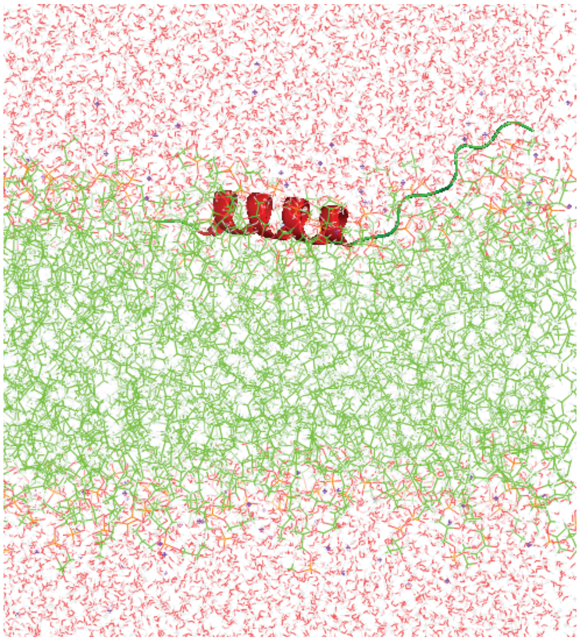

Initial MD snapshot **Final MD snapshot**
