# Supplementary material for: Membrane Mediated Antimicrobial and Antitumor Activity of Cathelicidin 6: Structural Insights from Molecular Dynamics Simulation on Multi-Microsecond Scale
Source: PLoS One. 2016 Jul 8;11(7):e0158702. doi: 10.1371/journal.pone.0158702 (PMC4938549; doi:10.1371/journal.pone.0158702)
Supplement: S6 Fig — The peptide molecules are colored as blue, red, green and black, and the lipid molecules as cyan for the three different heterogeneous membrane systems (TLM, LLM and hyPLM). The anionic lipid molecules are shown in cyan, and yellow represents the peptide C-terminal residues. All coarse grained beads are represented in VDW format. Selective aggregations of BMAP27 surrounding anionic lipids are indicated by arrows. (PDF) [file pone.0158702.s006.pdf]

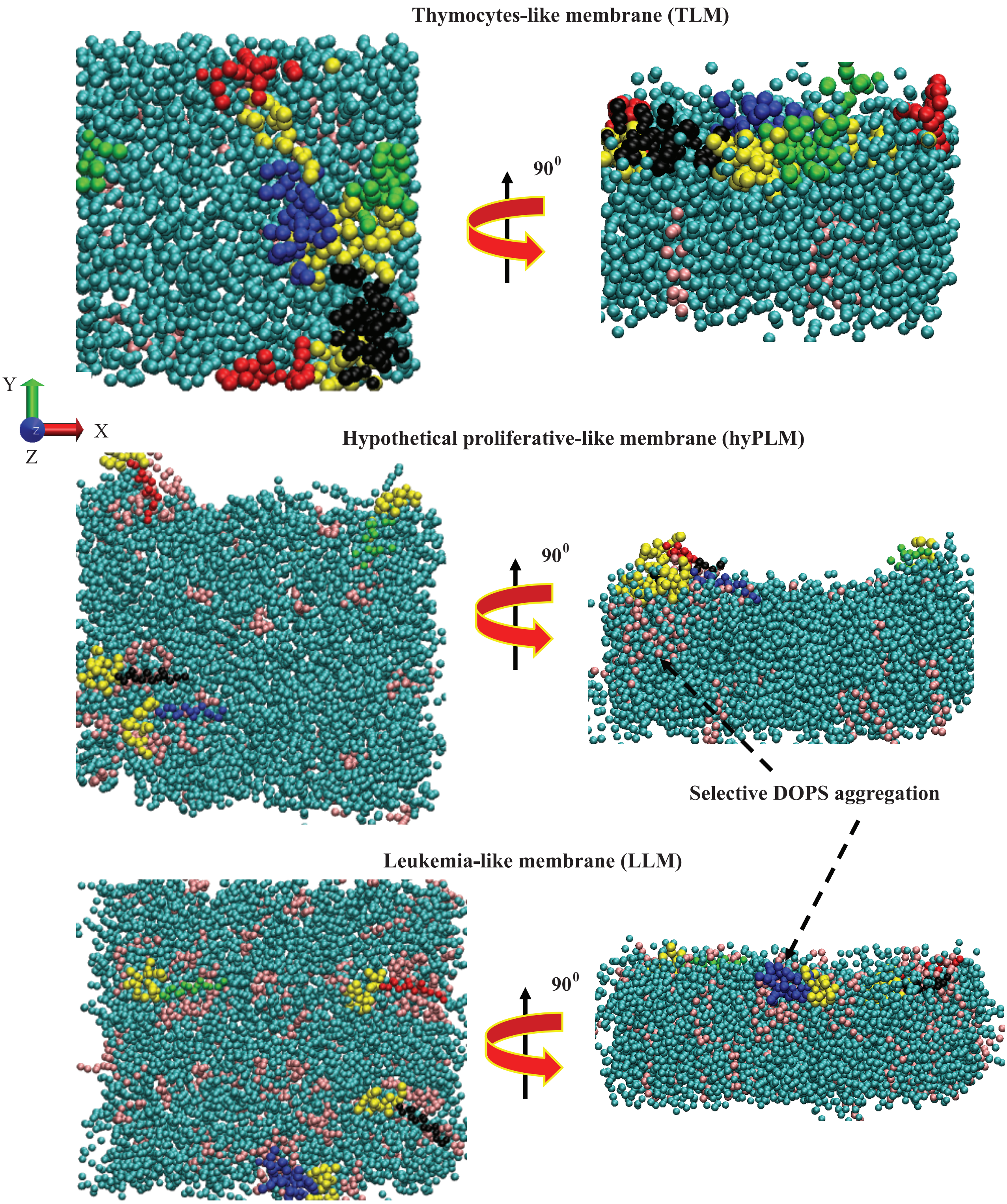

Thymocytes-like membrane (TLM)
90⁰
Y
X
Z
Hypothetical proliferative-like membrane (hyPLM)
90⁰
Selective DOPS aggregation
Leukemia-like membrane (LLM)
90⁰
