# Supplementary material for: Membrane Mediated Antimicrobial and Antitumor Activity of Cathelicidin 6: Structural Insights from Molecular Dynamics Simulation on Multi-Microsecond Scale
Source: PLoS One. 2016 Jul 8;11(7):e0158702. doi: 10.1371/journal.pone.0158702 (PMC4938549; doi:10.1371/journal.pone.0158702)
Supplement: S1 Table — (DOCX) [file pone.0158702.s009.docx]

**S1 Table. Effect of BMAP27 on the lateral diffusion (D_L_) of lipid molecules in CG-MD models.**

| **CG-system** | **Total time (µs)** | **D_L_ in cm^2^ s^-1^ (initial)** | **D_L_ in cm^2^ s^-1^ (final)** | **Remark** |
| --- | --- | --- | --- | --- |
| **DPPC** | 10 | 0.027 (± 0.002) x 10^-5^ | 0.018 (± 0.006) x 10^-5^ | Significant |
| **DOPS** | 10 | 0.052 (± 0.005) x 10^-5^ | 0.037 (± 0.008) x 10^-5^ | Significant |
| **DOPG** | 10 | 0.030 (± 0.003) x 10^-5^ | 0.025 (± 0.003) x 10^-5^ | Significant |
| **TLM** | 20 | 0.010 (± 0.001) x 10^-5^ | 0.011 (± 0.003) x 10^-5^ | Insignificant |
| **hyPLM** | 20 | 0.014 (± 0.005) x 10^-5^ | 0.014 (± 0.002) x 10^-5^ | Insignificant |
| **LLM** | 20 | 0.018 (± 0.001) x 10^-5^ | 0.020 (± 0.001) x 10^-5^ | Insignificant |
| **Micelle-like oligomer** | 10 | 0.028 (± 0.001) x 10^-5^ | 0.017 (± 0.001) x 10^-5^ | Significant |
| **End-to-end oligomer** | 13 | 0.018 (± 0.001) x 10^-5^ | 0.020 (± 0.001) x 10^-5^ | Insignificant |

TLM, thymocytes-like membrane; LLM, leukemia-like membrane; hyPLM: hypothetical proliferative-like membrane.
